# Supplementary material for: Sesquiterpenes from the Fungus Antrodiella albocinnamomea with Cytotoxicity and Antibacterial Activity
Source: J Fungi (Basel). 2023 Apr 28;9(5):521. doi: 10.3390/jof9050521 (PMC10219565; doi:10.3390/jof9050521)
Supplement: Supplementary file 1 [file jof-09-00521-s001.zip › Supporting information_JOF.pdf]

Supporting Information for

Sesquiterpenes from the Fungus *Antrodiella albocinnamomea* with Cytotoxicity and Antibacterial Activity

Jin-Lei Ning †, Feng Wu †, Ji-Kai Liu, Juan He * and Tao Feng *

School of Pharmaceutical Sciences, South-Central Minzu University, Wuhan 430074, China; jlning026@163.com (J.-L.N.); wfeng828@163.com (F.W.); jkliu@mail.kib.ac.cn (J.-K.L.)

* Correspondence: 2015048@mail.scuec.edu.cn (J.H.); tfeng@mail.scuec.edu.cn (T.F.)

† These authors contributed equally to this work.

Content

Sections S1. Computational details

S1.1 Computational details for albocinnamin A (1a/1b) (ECD)

S1.2 Computational details for albocinnamin B (2) (ECD)

S1.3 Computational details for albocinnamin C (3) (ECD)

S1.4 Computational details for albocinnamin F (6) (ECD)

S1.5 Computational details for albocinnamin G (7) (ECD)

Sections S2. X-Ray details

S2.1 X-Ray details for albocinnamin A (1a/1b)

S2.1 X-Ray details for albocinnamin E (5)

Sections S3. Supplementary of NMR and HRESIMS

S3.1 NMR and HRESIMS spectra of albocinnamin A (1a/1b)

S3.2 NMR and HRESIMS spectra of albocinnamin B (2)

S3.3 NMR and HRESIMS spectra of albocinnamin C (3)

S3.4 NMR and HRESIMS spectra of albocinnamin D (4)

S3.5 NMR and HRESIMS spectra of albocinnamin E (5)

S3.6 NMR and HRESIMS spectra of albocinnamin F (6)

S3.7 NMR and HRESIMS spectra of albocinnamin G (7)

S3.8 NMR and HRESIMS spectra of albocinnamin H (8)

Section S1. ECD calculations for 1−3, 6−7

S1.1. Computational details for 1a/1b (ECD)

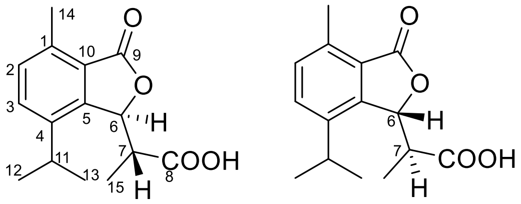

(+)-**1a** 6*S*,7*R* (−)-**1b** 6*R*,7*S*

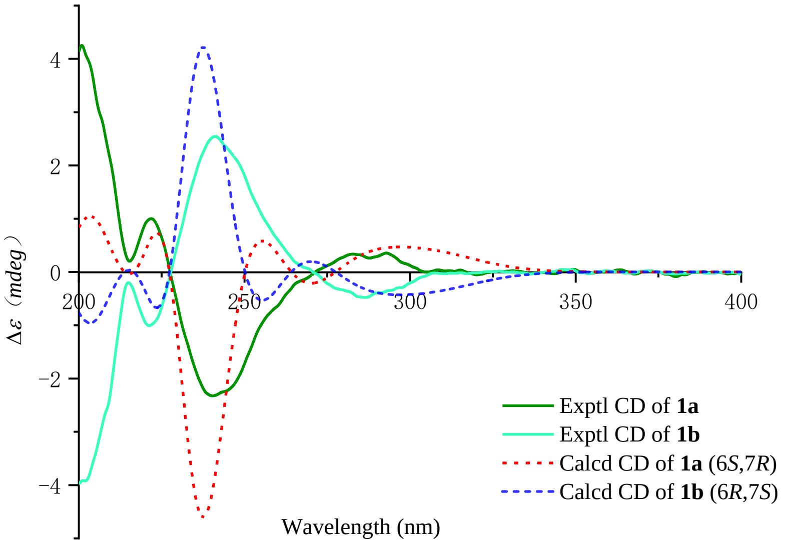

Figure S1. Comparison of the calculated ECD spectra for possible structure with the experimental spectrum of **1a**/**1b** in methanol with PCM model.

Table S1. Energy analysis for conformers of **1a** (6*S*,7*R*) and **1b** (6*R*,7*S*) at B3LYP/6-311G (d, p) level in the gas phase

Species	*E′=E+ZPE*	*E*	*H*	*G*	*ΔG(kcal/mol)*	*ΔE(kcal/mol)*	*PE%*
6*R*7*S*-1.out:	-883.357250	-883.338715	-883.337770	-883.403363	0.000000	0.000000	96.34%
6*R*7*S*-2.out:	-883.353359	-883.334947	-883.334002	-883.399681	0.003682	2.310490	1.95%
6*R*7*S*-3.out:	-883.351231	-883.332558	-883.331614	-883.397796	0.005567	3.493345	0.26%
6*R*7*S*-4.out:	-883.351128	-883.332434	-883.331490	-883.398227	0.005136	3.222889	0.42%
6*R*7*S*-5.out:	-883.353294	-883.334829	-883.333885	-883.399047	0.004316	2.708331	0.99%
6*R*7*S*-6.out:	-883.349127	-883.330324	-883.329380	-883.395961	0.007402	4.644825	0.04%
6*S*7*R*-1.out:	-883.357250	-883.338715	-883.337770	-883.403363	0.000000	0.000000	96.91%
6*S*7*R*-2.out:	-883.353359	-883.334947	-883.334002	-883.399681	0.003682	2.310490	1.96%
6*S*7*R*-3.out:	-883.351231	-883.332558	-883.331614	-883.397796	0.005567	3.493345	0.27%
6*S*7*R*-4.out:	-883.351127	-883.332434	-883.331490	-883.398220	0.005143	3.227281	0.42%
6*S*7*R*-5.out:	-883.349127	-883.330324	-883.329380	-883.395961	0.007402	4.644825	0.04%
6*S*7*R*-6.out:	-883.351128	-883.332434	-883.331490	-883.398225	0.005138	3.224144	0.42%

E, *E′*, *H*, *G*: total energy, total energy with zero-point energy (ZPE), enthalpy, and Gibbs free energy

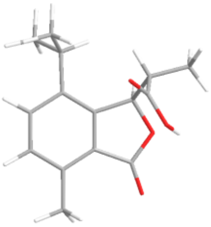

6*R*7*S*-1

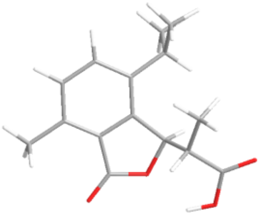

6*R*7*S*-2

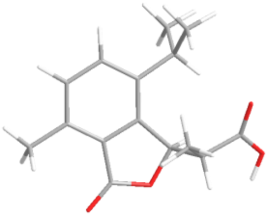

6*R*7*S*-3

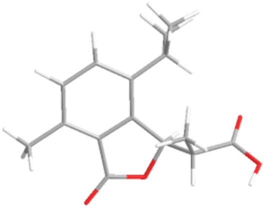

6*R*7*S*-4

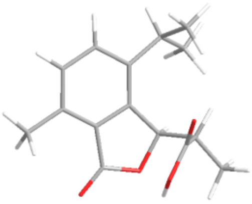

6*R*7*S*-5

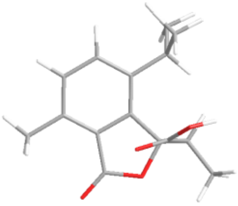

6*R*7*S*-6

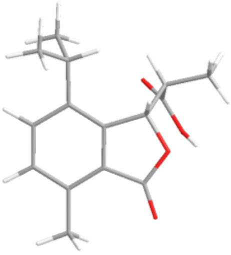

6*S*7*R*-1

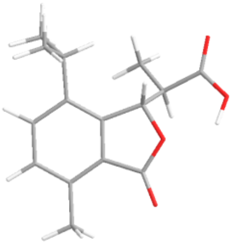

6*S*7*R*-2

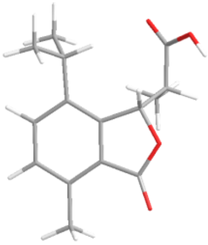

6*S*7*R*-3

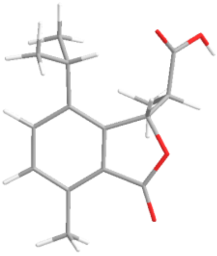

6*S*7*R*-4

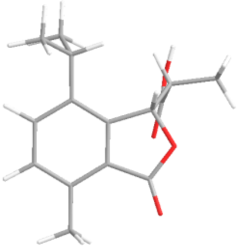

6*S*7*R*-5

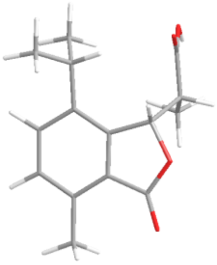

6*S*7*R*-6

Figure S2. Main conformers of 6*R*7*S* and 6*S*7*R*

S1.2. Computational details for 2 (ECD)

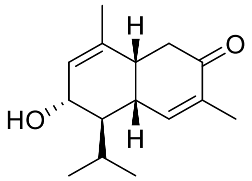

3*S*,4*R*,5*S*,10*R*

2

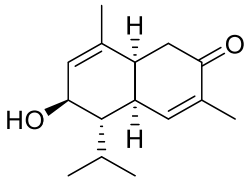

3*R*,4*S*,5*R*,10*S*

2'

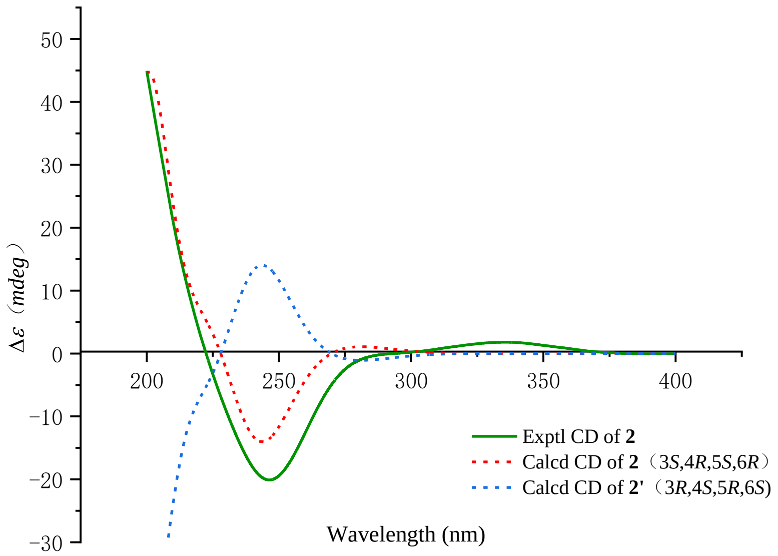

Figure S3. Comparison of the calculated ECD spectra for possible structure with the experimental spectrum of **2** in methanol with PCM model.

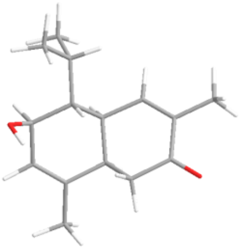

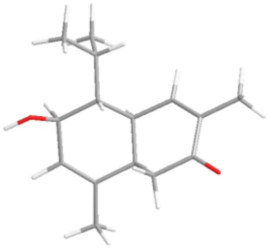

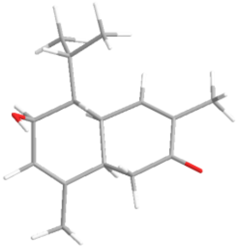

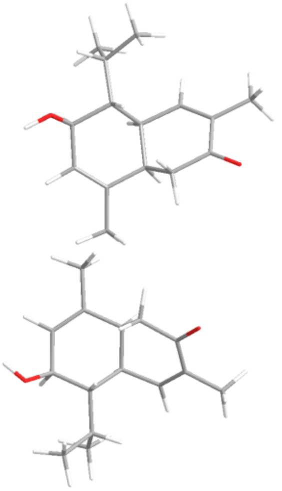

2-1 **2**-2 **2**-3 **2**-4

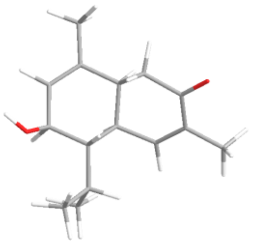

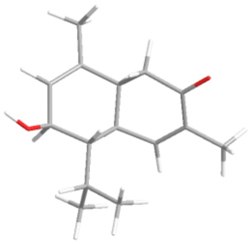

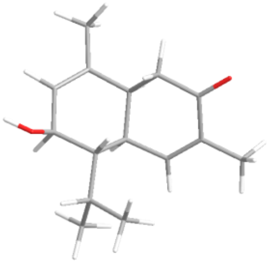

2'-1 **2'**-2 **2'**-3 **2'**-4

Figure S4. Main conformers of 2 and 2'

S1.3. Computational details for 3 (ECD)

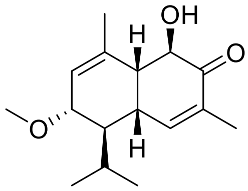

3*S*,4*R*,5*R*,9*R*,10*R*

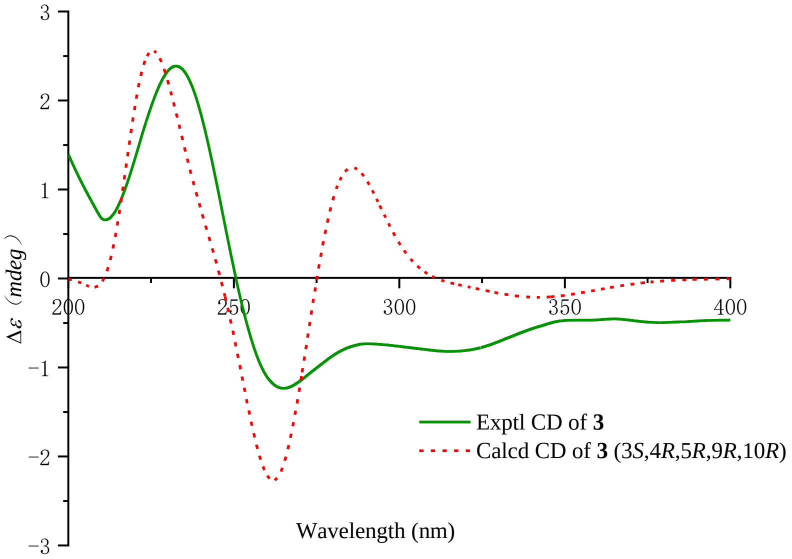

Figure S5. Comparison of the calculated ECD spectra for possible structure with the experimental spectrum of **3** in methanol with PCM model.

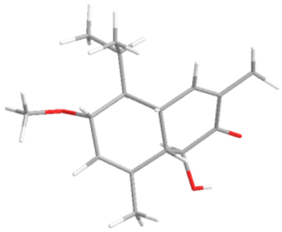

3*S*4*R*5*R*9*R*10*R*-1

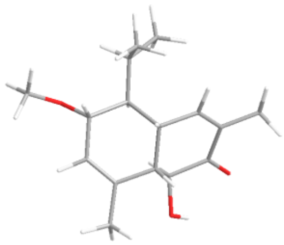

3*S*4*R*5*R*9*R*10*R*-2

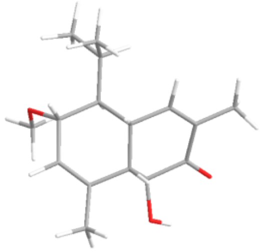

3*S*4*R*5*R*9*R*10*R*-3

Figure S6. Main conformers of 3*S*4*R*5*R*9*R*10*R*.

Table S2. Energy analysis for conformers of **2** (3*S*4*R*5*S*10*R*) at B3LYP/6-311G (d, p) level in the gas phase

Species	*E′=E+ZPE*	*E*	*H*	*G*	*ΔG(kcal/mol)*	*ΔE(kcal/mol)*	*PE%*
3*S*4*R*5*S*10*R*-1.out:	-735.005251	-734.987719	-734.986775	-735.049735	0.000000	0.000000	57.81%
3*S*4*R*5*S*10*R*-2.out:	-735.002565	-734.984907	-734.983962	-735.047466	0.002269	1.423819	5.22%
3*S*4*R*5*S*10*R*-3.out:	-735.005259	-734.987755	-734.986811	-735.049240	0.000495	0.310617	34.21%
3*S*4*R*5*S*10*R*-4.out:	-735.002691	-734.985047	-734.984103	-735.046862	0.002873	1.802835	2.75%
3*R*4*S*5*R*10*S*-1.out:	-734.062317	-734.044817	-734.043873	-734.106993	0.000000	0.000000	72.05%
3*R*4*S*5*R*10*S*-2.out:	-734.060257	-734.042690	-734.041746	-734.104736	0.002257	1.416289	6.59%
3*R*4*S*5*R*10*S*-3.out:	-734.061789	-734.044317	-734.043373	-734.105712	0.001281	0.803840	18.54%
3*R*4*S*5*R*10*S*-4.out:	-734.059816	-734.042206	-734.041261	-734.103939	0.003054	1.916414	2.83%

Table S3. Energy analysis for conformers of **3** (3*S*4*R*5*R*9*R*10*R*) at B3LYP/6-311G (d, p) level in the gas phase

Species	*E′=E+ZPE*	*E*	*H*	*G*	*ΔG(kcal/mol)*	*ΔE(kcal/mol)*	*PE%*
3*S*4*R*5*R*9*R*10*R*-1.out:	848.563791	-848.544292	-848.543348	-848.610356	0.001450	0.909889	11.32%
3*S*4*R*5*R*9*R*10*R*-2.out:	848.564319	-848.544504	-848.543559	-848.611448	0.000358	0.224648	36.03%
3*S*4*R*5*R*9*R*10*R*-3.out:	848.564766	-848.545156	-848.544212	-848.611806	0.000000	0.000000	52.65%

E, *E′*, *H*, *G*: total energy, total energy with zero-point energy (ZPE), enthalpy, and Gibbs free energy

S1.4. Computational details for 6 (ECD)

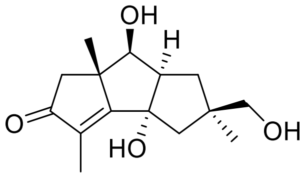

1*S*,6*S*,7*S*,8*R*,10*R*

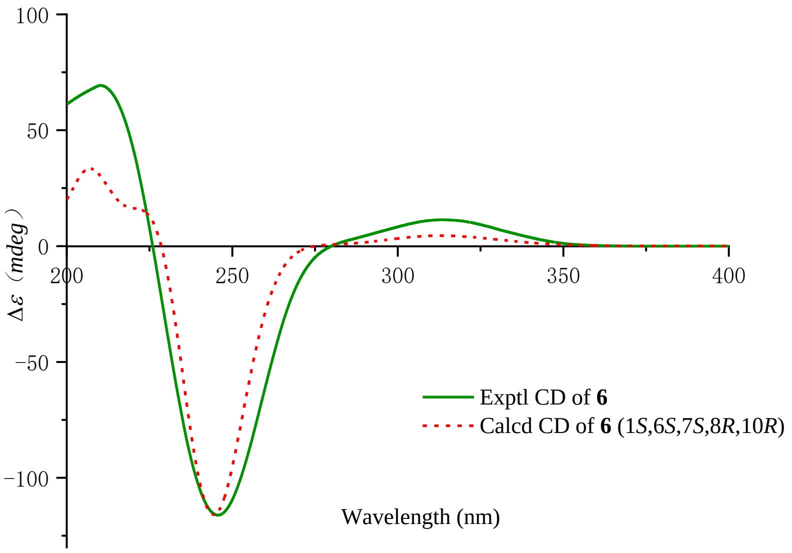

Figure S7. Comparison of the calculated ECD spectra for possible structure with the experimental spectrum of 6 in methanol with PCM model

Table S4. Energy analysis for conformers of **6** (6*S*7*S*8*S*10*R*) at B3LYP/6-311G (d, p) level in the gas phase

Species	*E′=E+ZPE*	*E*	*H*	*G*	*ΔG(kcal/mol)*	*ΔE(kcal/mol)*	*PE%*
1S6S7S8R10R-1.out:	885.430857	-885.411688	-885.410744	-885.476233	0.003444	2.161143	1.24%
1S6S7S8R10R-2.out:	885.432722	-885.413560	-885.412616	-885.477981	0.001696	1.064256	7.91%
1S6S7S8R10R-3.out:	885.434557	-885.415464	-885.414519	-885.479677	0.000000	0.000000	47.73%
1S6S7S8R10R-4.out:	885.430334	-885.411161	-885.410217	-885.475683	0.003994	2.506273	0.69%
1S6S7S8R10R-5.out:	885.430688	-885.411505	-885.410561	-885.476070	0.003607	2.263427	1.04%
1S6S7S8R10R-6.out:	885.431272	-885.412181	-885.411237	-885.476612	0.003065	1.923317	1.85%
1S6S7S8R10R-7.out:	885.432170	-885.413078	-885.412134	-885.477358	0.002319	1.455195	4.09%
1S6S7S8R10R-8.out:	885.429418	-885.410209	-885.409264	-885.474934	0.004743	2.976278	0.31%
1S6S7S8R10R-9.out:	885.433371	-885.414377	-885.413433	-885.478403	0.001274	0.799447	12.37%

Table S5. Energy analysis for conformers of **7** (6*S*7*S*8*S*10*R*) at B3LYP/6-311G (d, p) level in the gas phase

Species	*E′=E+ZPE*	*E*	*H*	*G*	*ΔG(kcal/mol)*	*ΔE(kcal/mol)*	*PE%*
6S7S8S10R-1.out:	-808.009663	-807.991905	-807.990961	-808.054232	0.000000	0.000000	47.73%
6S7S8S10R-2.out:	-808.006822	-807.989028	-807.988084	-808.051349	0.002883	1.809110	2.25%
6S7S8S10R-3.out:	-808.009300	-807.991625	-807.990680	-808.053428	0.000804	0.504518	20.36%
6S7S8S10R-4.out:	-808.007265	-807.989392	-807.988448	-808.052180	0.002052	1.287649	5.42%
6S7S8S10R-5.out:	-808.009488	-807.991824	-807.990880	-808.053593	0.000639	0.400979	24.25%

E, *E′*, *H*, *G*: total energy, total energy with zero-point energy (ZPE), enthalpy, and Gibbs free energy

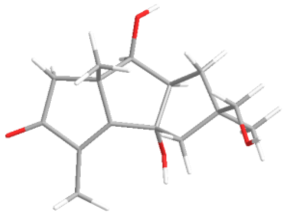

1*S*6*S*7*S*8*R*10*R*-1

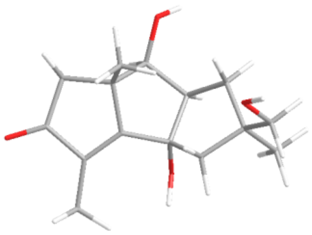

1*S*6*S*7*S*8*R*10*R*-2

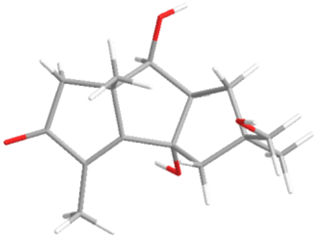

1*S*6*S*7*S*8*R*10*R*-3

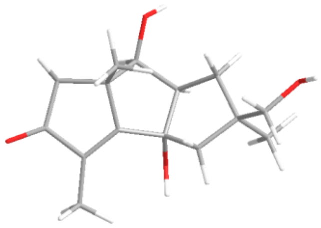

1*S*6*S*7*S*8*R*10*R*-4

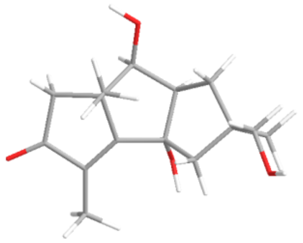

1*S*6*S*7*S*8*R*10*R*-5

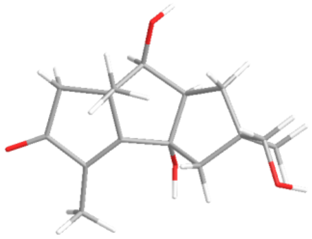

1*S*6*S*7*S*8*R*10*R*-6

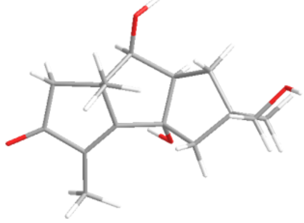

1*S*6*S*7*S*8*R*10*R*-7

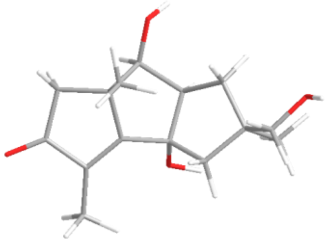

1*S*6*S*7*S*8*R*10*R*-8

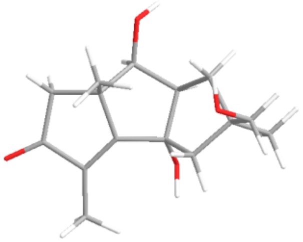

1*S*6*S*7*S*8*R*10*R*-9

Figure S8. Comparison of the calculated ECD spectra for possible structure with the experimental spectrum of 6 in methanol with PCM model

S1.5. Computational details for 7 (ECD)

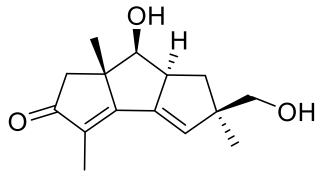

6*S*,7*S*,8*S*,10*R*

7

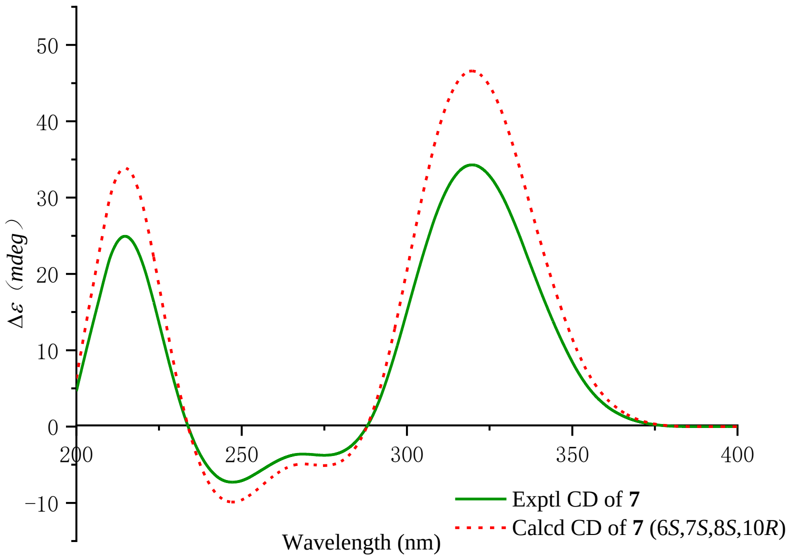

Figure S9. Comparison of the calculated ECD spectra for possible structure with the experimental spectrum of **7** in methanol with PCM model.

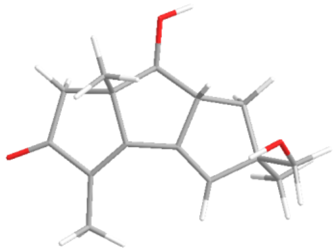

6*S*7*S*8*S*10*R*-1

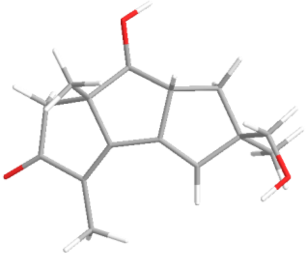

6*S*7*S*8*S*10*R*-2

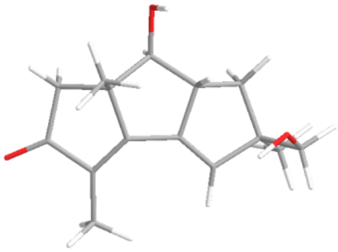

6*S*7*S*8*S*10*R*-3

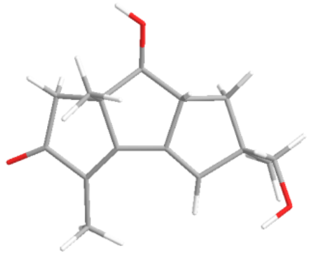

6*S*7*S*8*S*10*R*-4

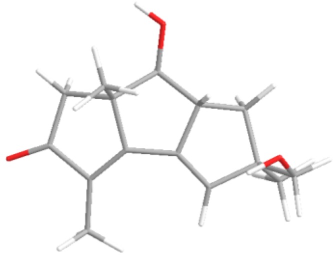

6*S*7*S*8*S*10*R*-5

Figure S10. Main conformers of 6*S*7*S*8*S*10*R*

Sections S2. X-Ray details

S2.1 X-Ray details for albocinnamin A (1a/1b)

Crystal data for compound **1a/1b**: approximate dimensions 0.600 mm x 0.130 mm x 0.110 mm, was used for the X-ray crystallographic analysis on the BRUKER D8 QUEST. $C_{15}H_{18}O_4$, (M = 262.29 g/mol): monoclinic, space group Pbca, a = 11.1153(5) Å, b = 13.4703(6) Å, c = 18.6151(8) Å, $\alpha = 90°$, $\beta = 90°$, $\gamma = 90°$, V = 2787.2(2) Å^3, Z = 8, T = 100(2) K, μ(Cu Kα) = 0.739 mm^{-1}, $Dcalc$ = 1.250 mg/m^3, 22765 reflections measured, 2749 independent reflections (R_{int} = 0.0608). The final R_1 was 0.0347 ($I > 2\sigma(I)$), and $wR(F^2)$ was 0.0923 ($I > 2\sigma(I)$). The final R_1 was 0.0456 (all data), and $wR(F^2)$ was 0.0962 (all data).

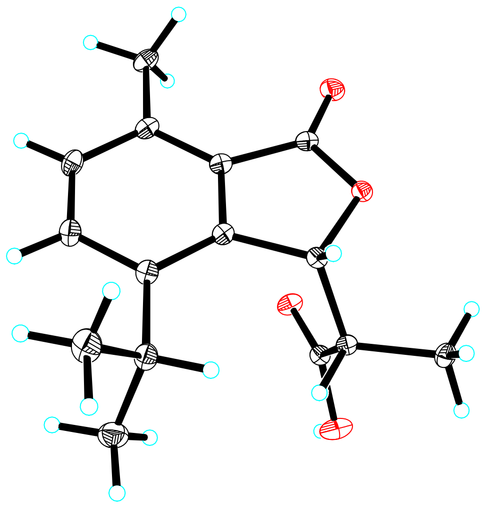

Figure S11. View of a molecule of **1a/1b** with the atom-labelling scheme.

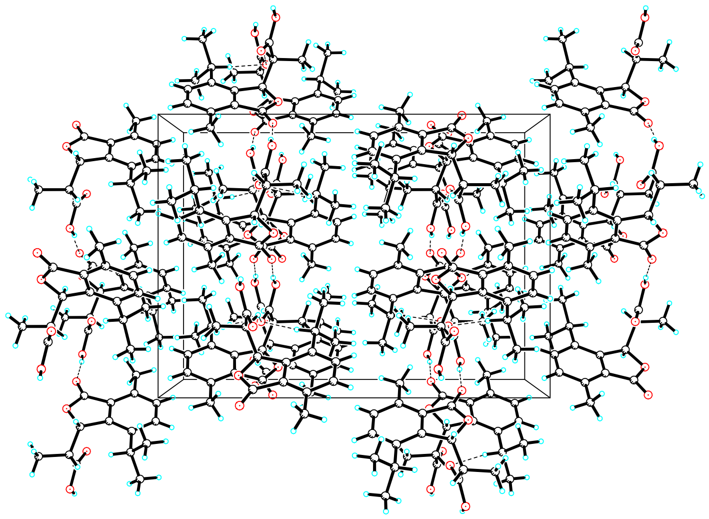

Figure S12. View of the pack drawing of **1a/1b**. Hydrogen-bonds are shown as dashed lines.

S2.2 X-Ray details for albocinnamin E (5)

Crystal data for compound **5**: approximate dimensions 0.820 mm x 0.570 mm x 0.440 mm, was used for the X-ray crystallographic analysis on the BRUKER D8 QUEST. $C_{15}H_{22}O_3$, (M = 250.32 g/mol): monoclinic, space group $P2_12_12_1$, a = 7.7864(4) Å, b = 9.0991(4) Å, c = 19.0099(9) Å, α = 90°, β = 90°, γ = 90°, V = 1346.84(11) Å^3, Z = 4, T = 100(2) K, μ(Cu Kα) = 0.676 mm^{-1}, D_{calc} = 1.235 mg/m^3, 12794 reflections measured, 2639 independent reflections (R_{int} = 0.0419). The final R_1 was 0.0309 ($I > 2\sigma(I)$), and $wR(F^2)$ was 0.0806 ($I > 2\sigma(I)$). The final R_1 was 0.0312 (all data), and $wR(F^2)$ was 0.0808 (all data). Flack parameter = 0.01(5).

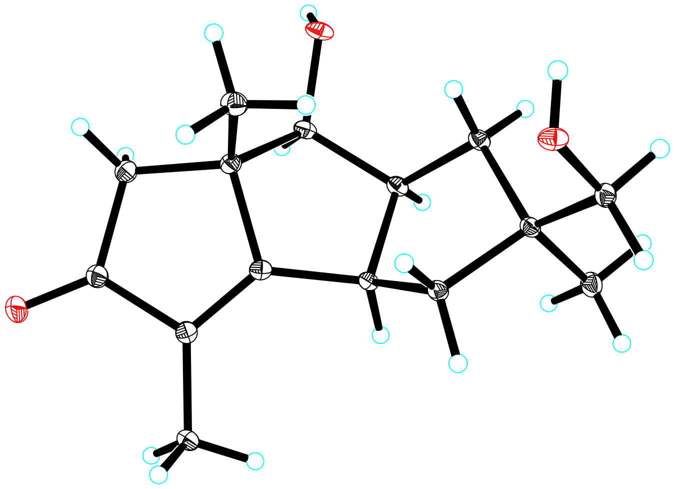

Figure S13. View of a molecule of **5** with the atom-labelling scheme.

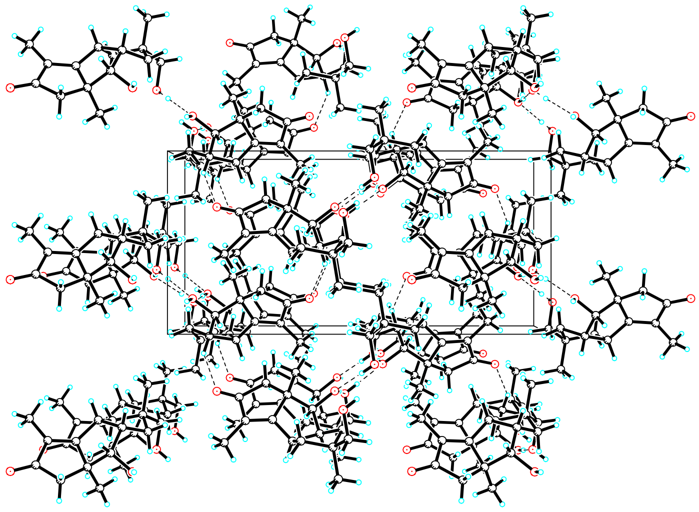

Figure S14. View of the pack drawing of **5**. Hydrogen-bonds are shown as dashed lines.

Sections S3. Supplementary of NMR and HRESIMS

S3.1 NMR and HRESIMS spectra of albocinnamin A (1a/1b)

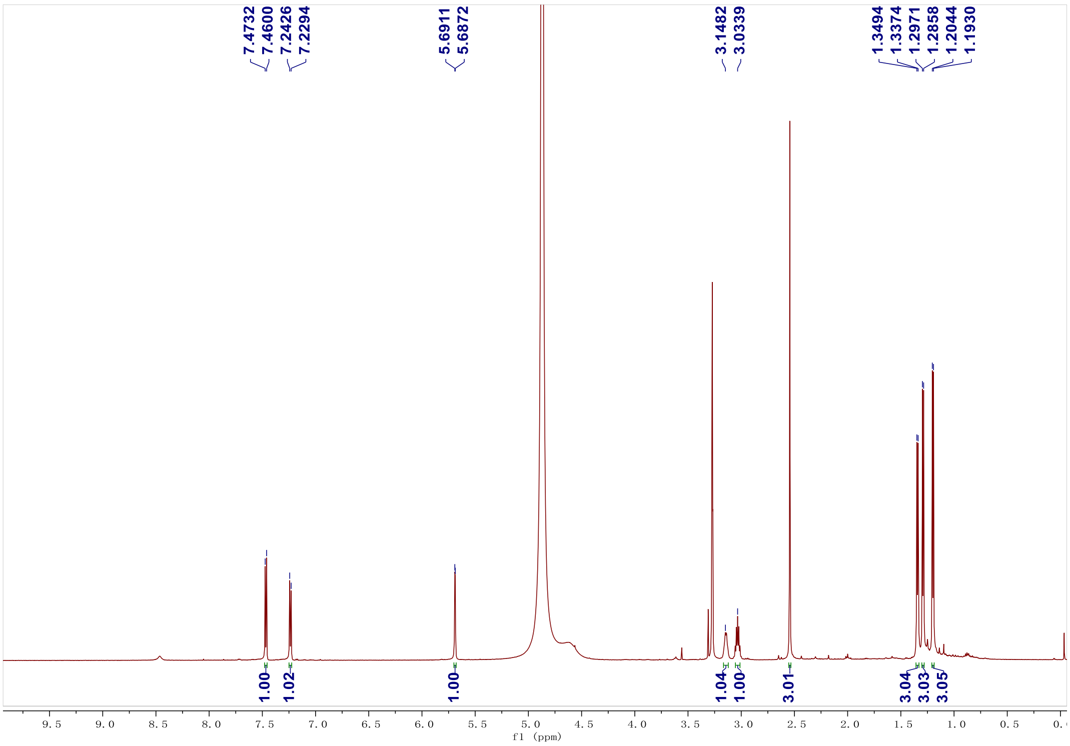

Figure S15. ¹H NMR spectrum of compound **1**. (600 MHz, methanol-*d*₄)

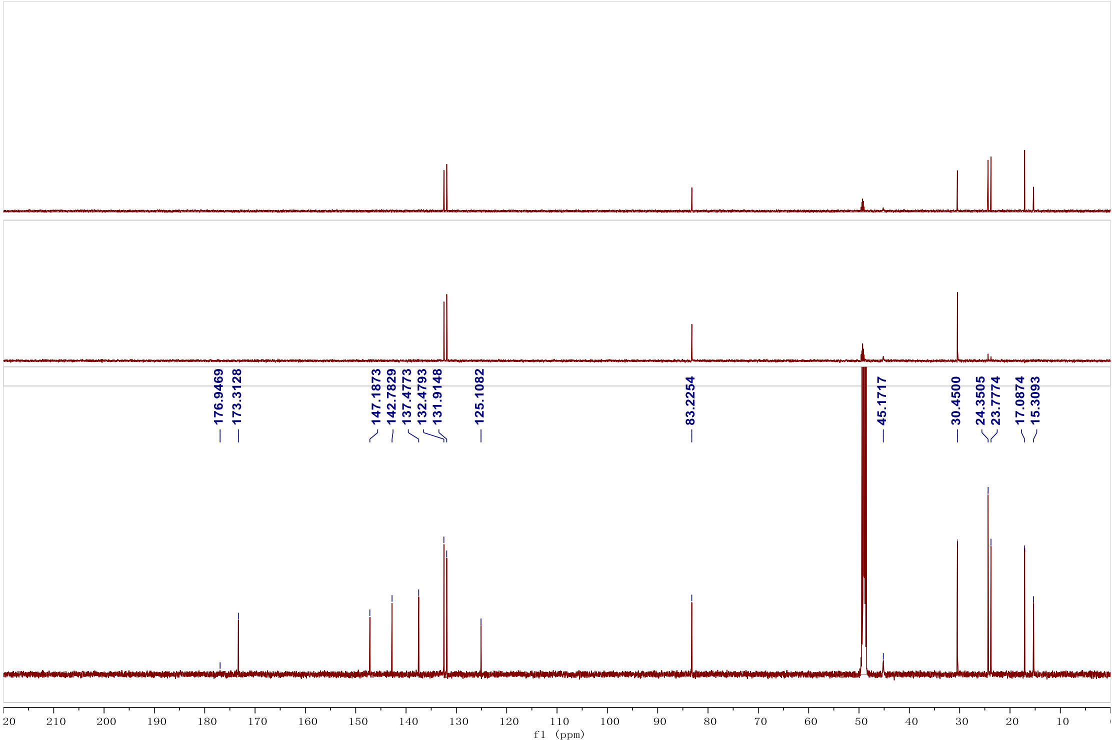

Figure S16. ¹³C NMR spectrum of compound **1**. (150 MHz, methanol-*d*₄)

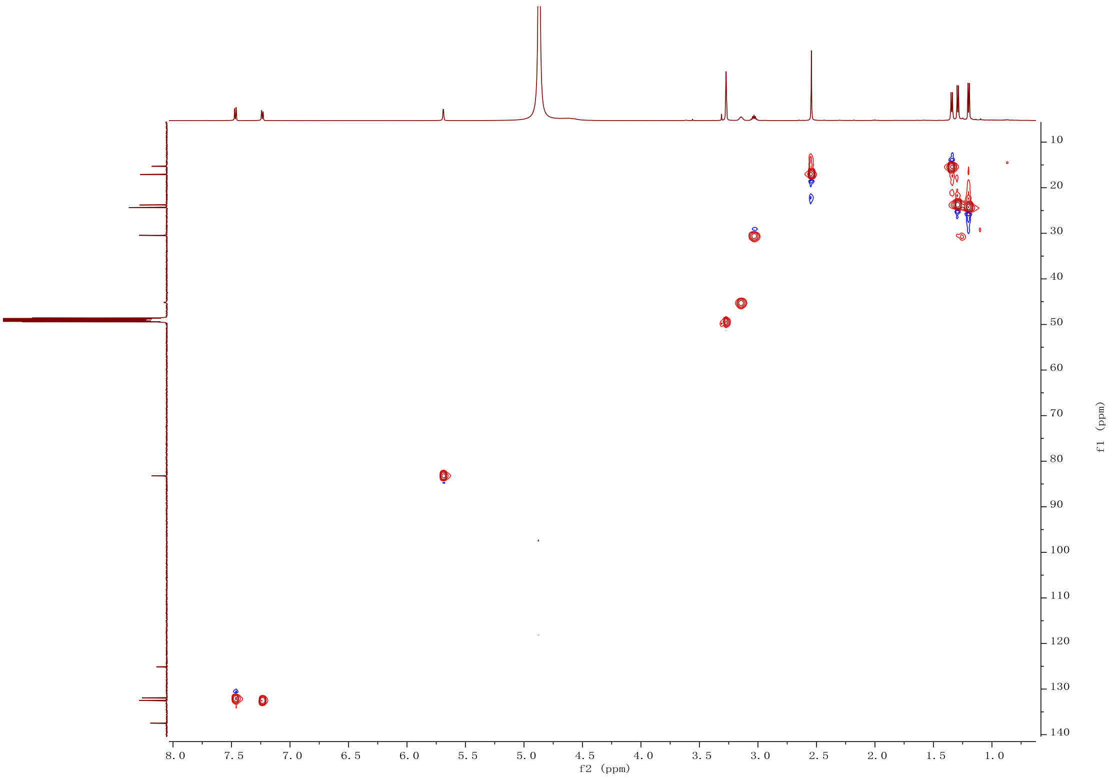

Figure S17. HSQC spectrum of compound **1**

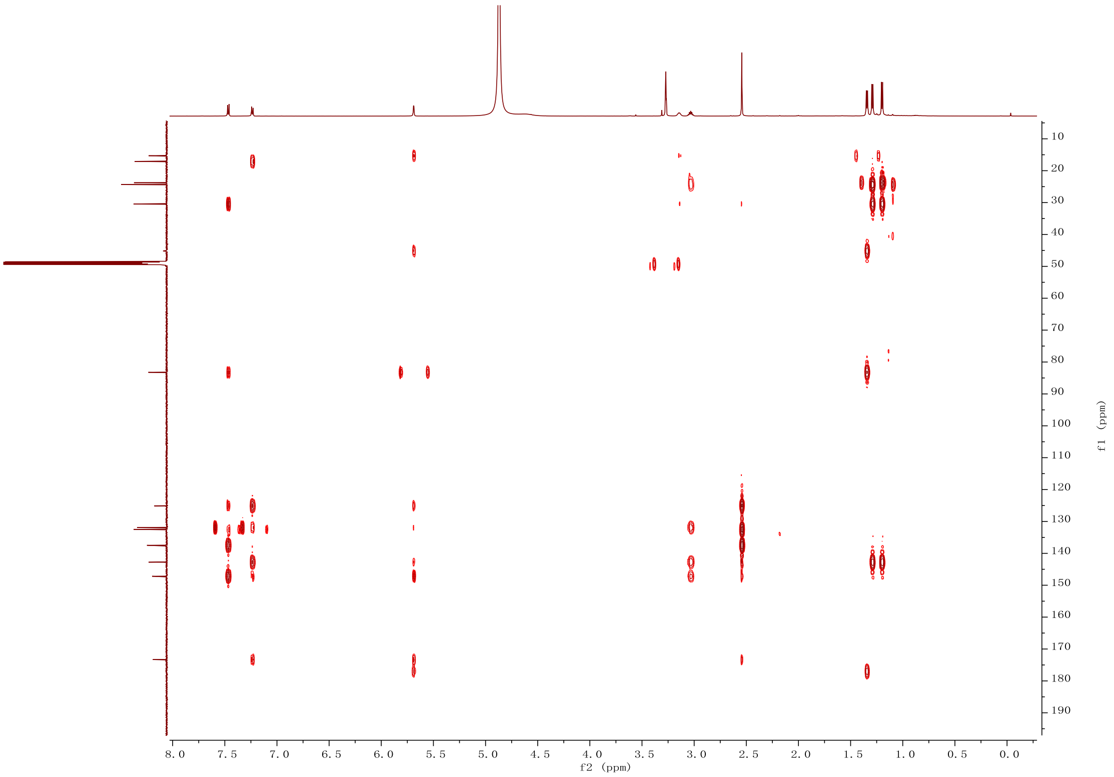

Figure S18.HMBC spectrum of compound **1**

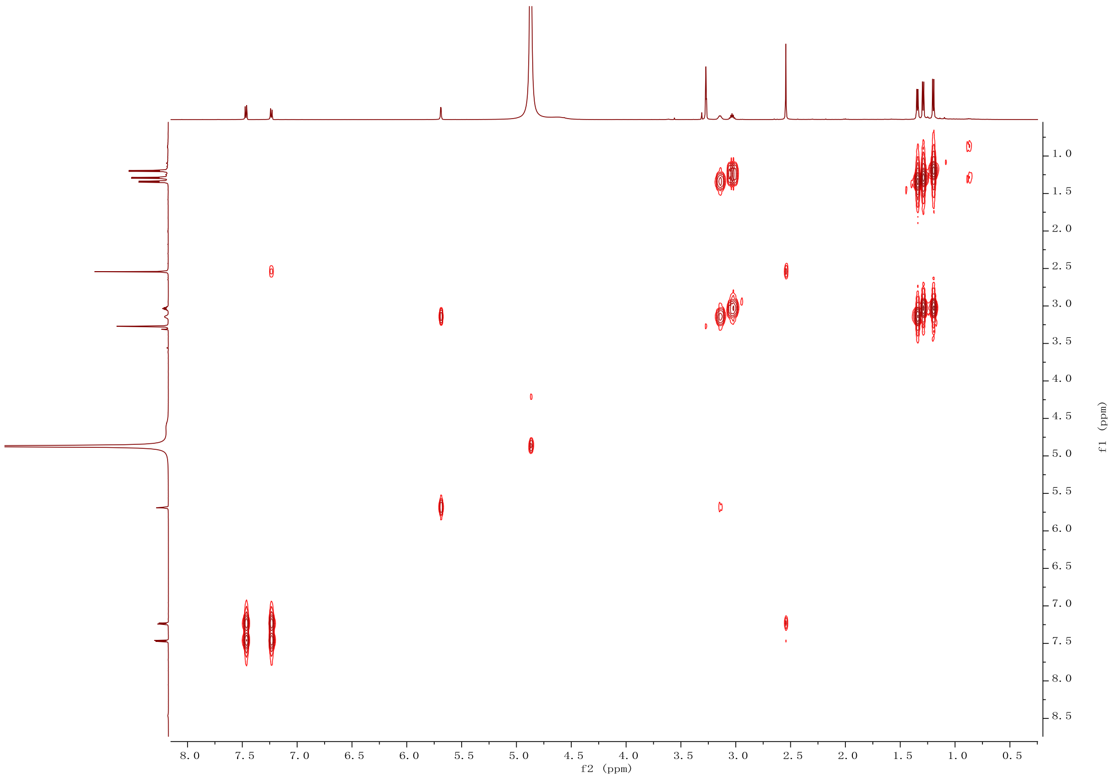

Figure S19. ^1H-^1H COSY spectrum of compound **1**

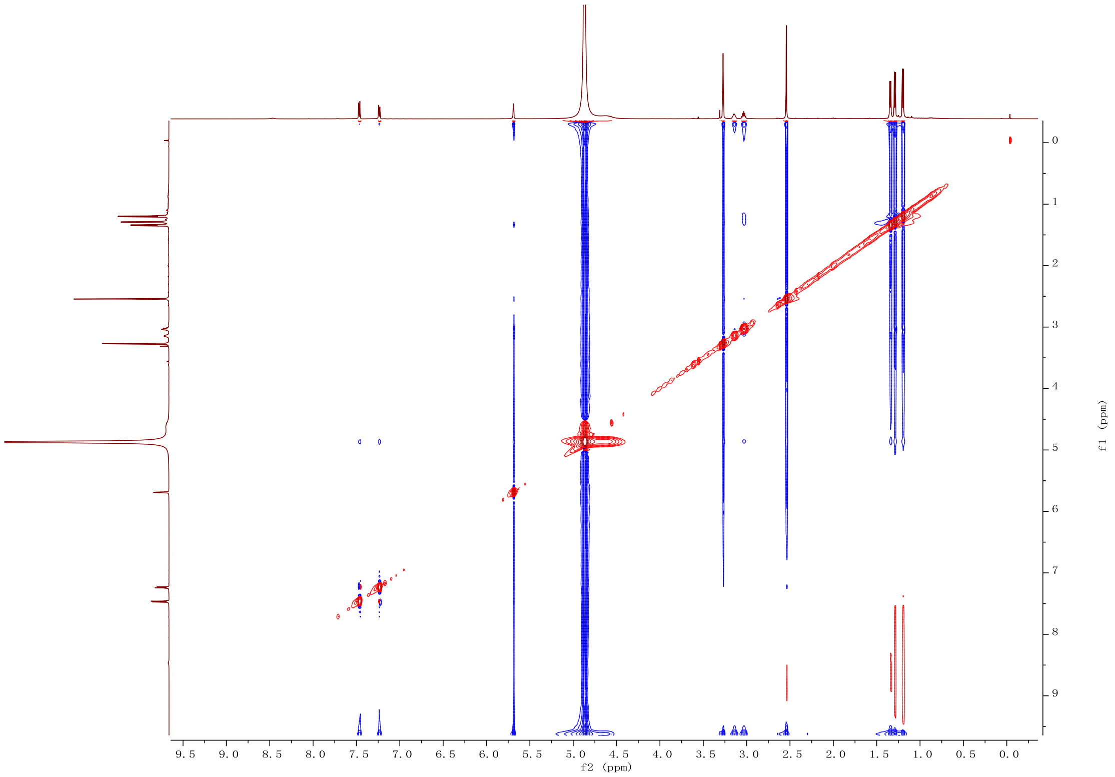

Figure S20. ROESY spectrum of compound **1**

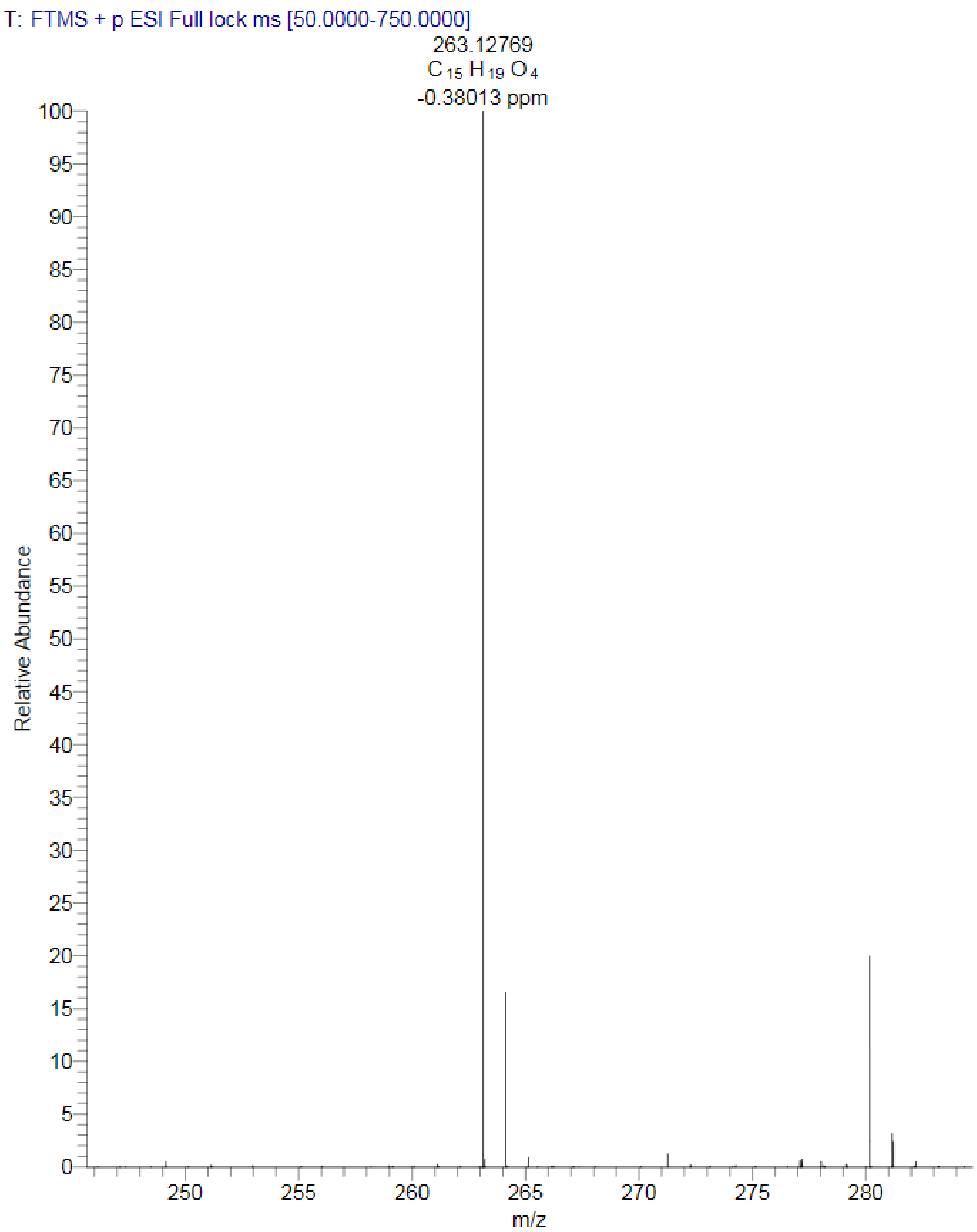

Figure S21. HRESIMS data of compound **1**

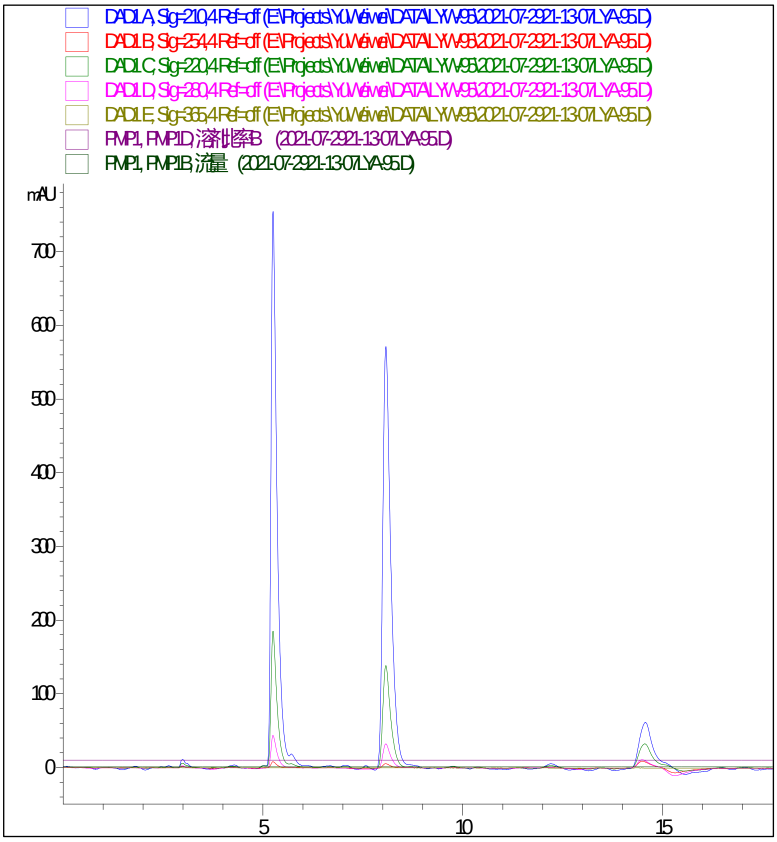

Figure S22. Chiral HPLC analysis of compound **1**

S3.2 NMR and HRESIMS spectra of albocinnamin B (2)

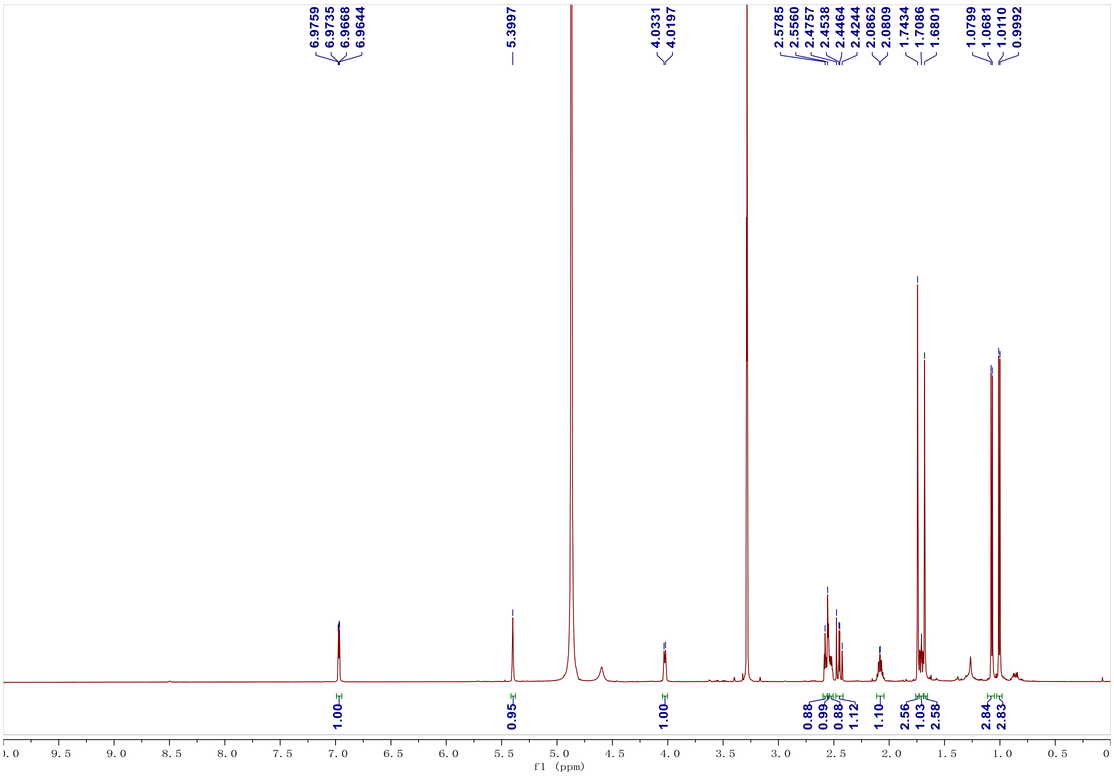

Figure S23. ^{1}H NMR spectrum of compound **2**. (600 MHz, methanol-d_4)

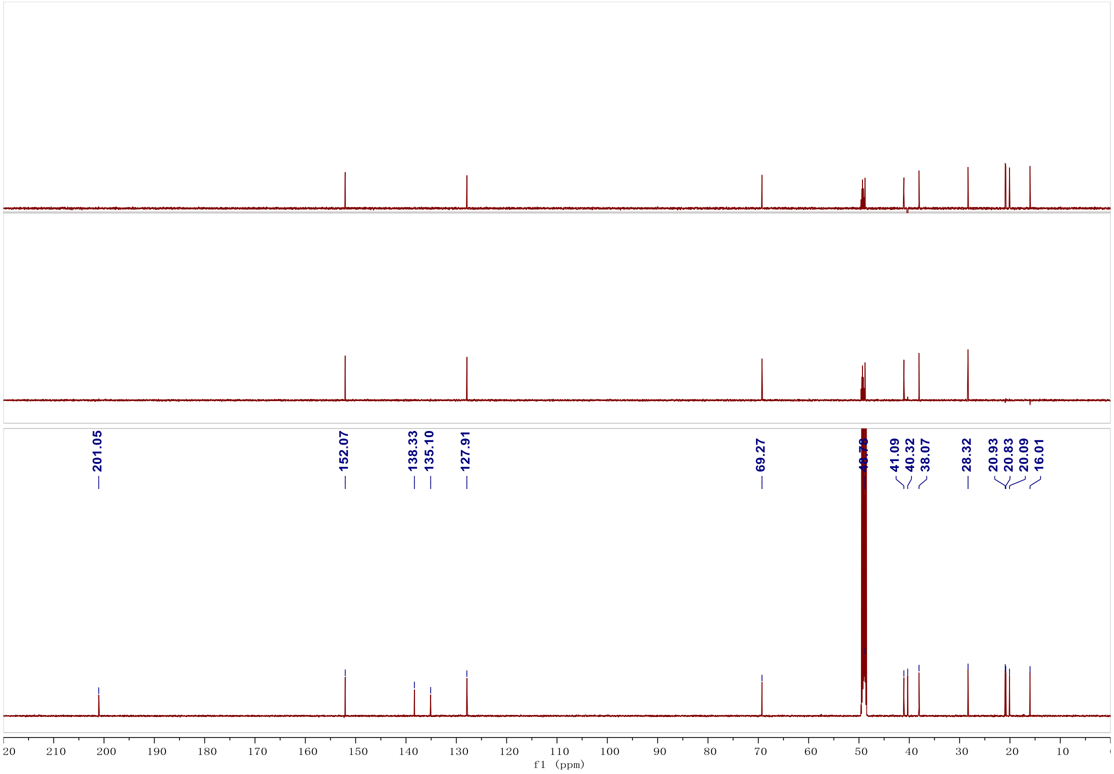

Figure S24. ^{13}C NMR spectrum of compound **2**. (150 MHz, methanol-d_4)

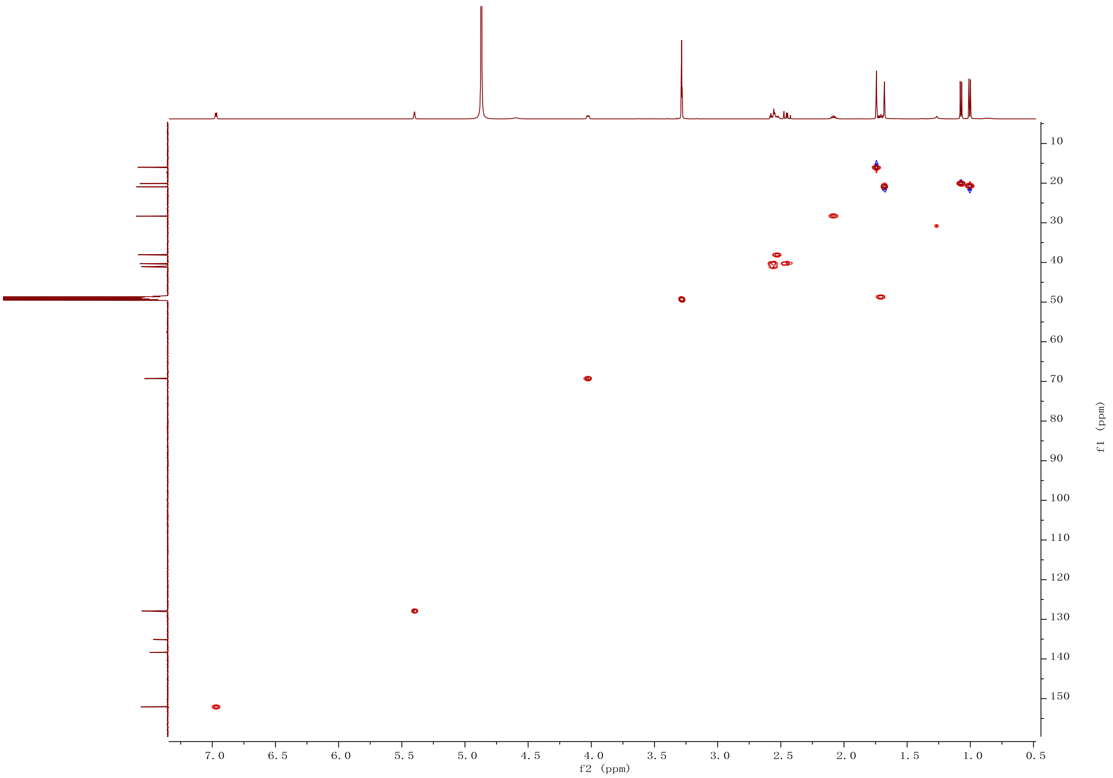

Figure S25. HSQC spectrum of compound **2**

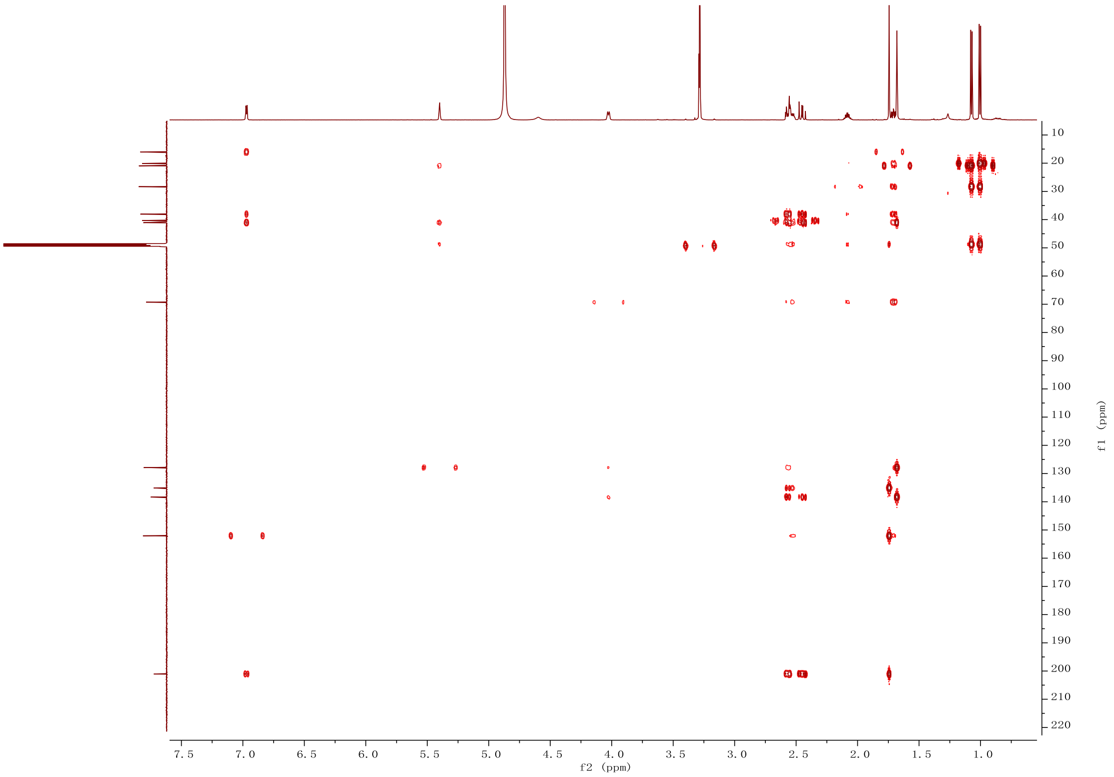

Figure S26. HMBC spectrum of compound **2**

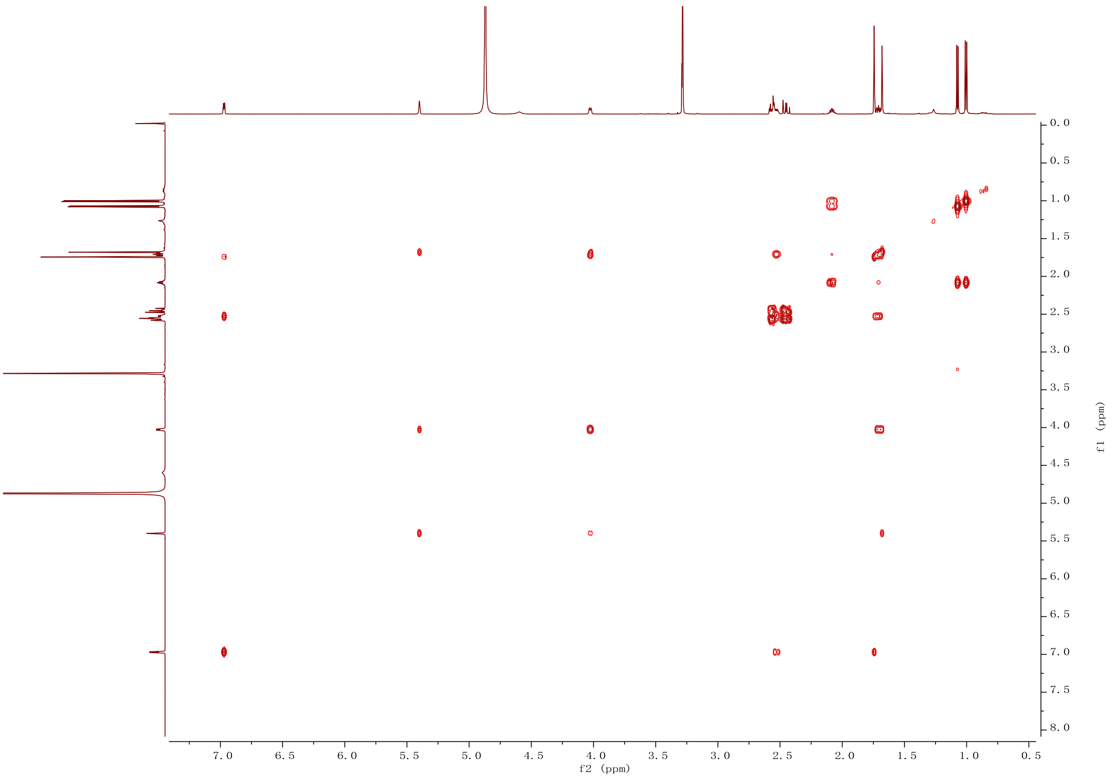

Figure S27. ^{1}H-^{1}H COSY spectrum of compound **2**

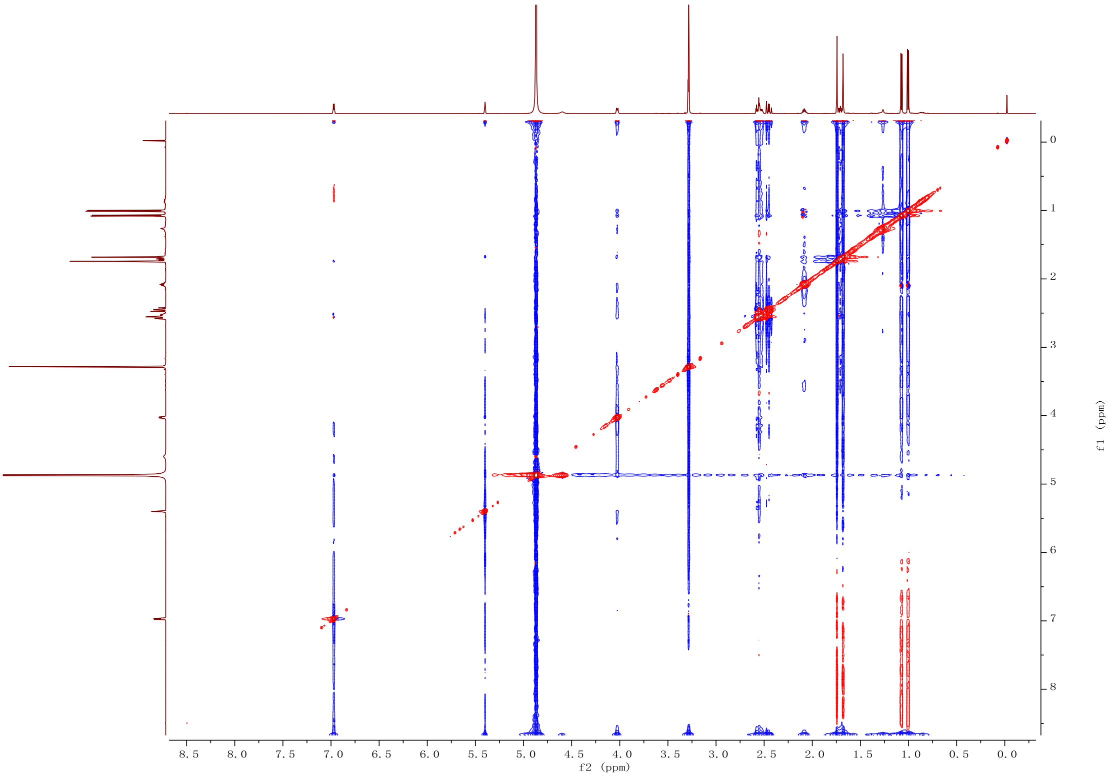

Figure S28. ROESY spectrum of compound **2**

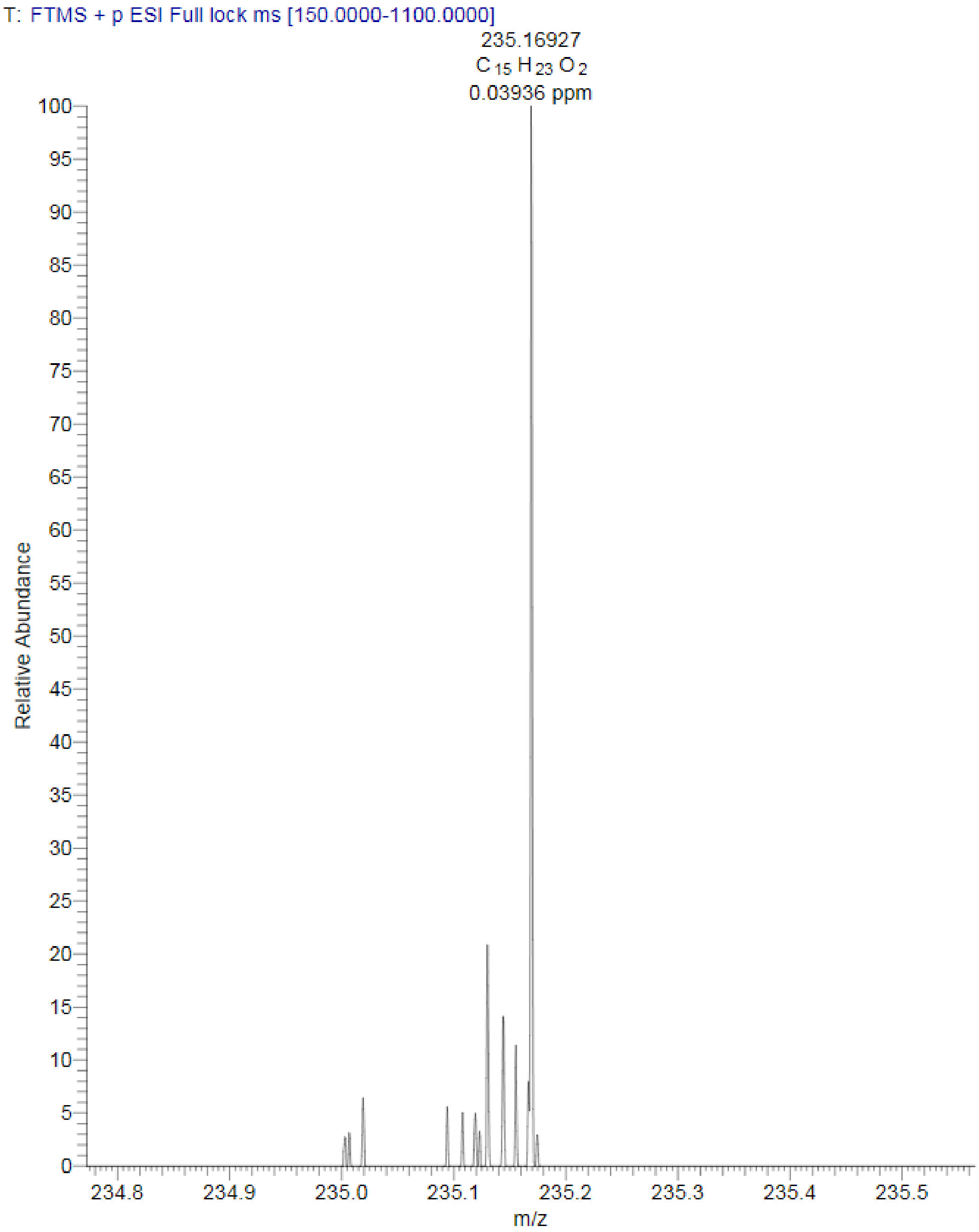

Figure S29. HRESIMS data of compound **2**

S3.3 NMR and HRESIMS spectra of albocinnamin C (3)

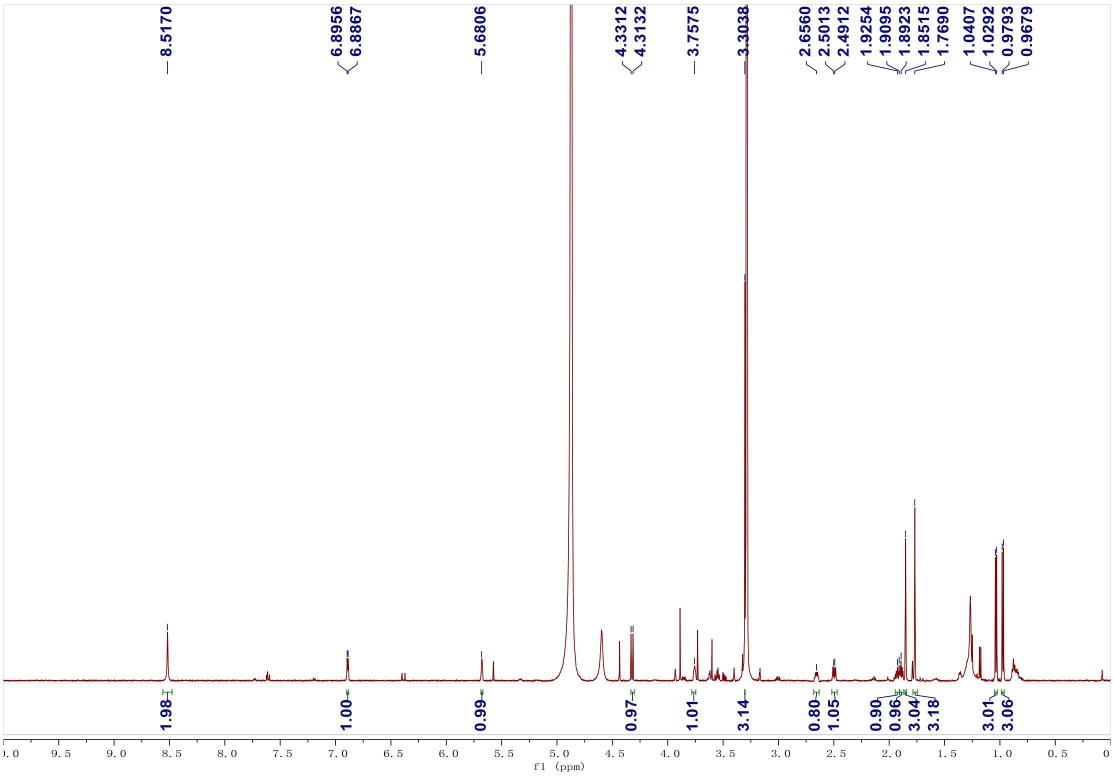

Figure S30. ^{1}H NMR spectrum of compound **3**. (600 MHz, methanol-d_4)

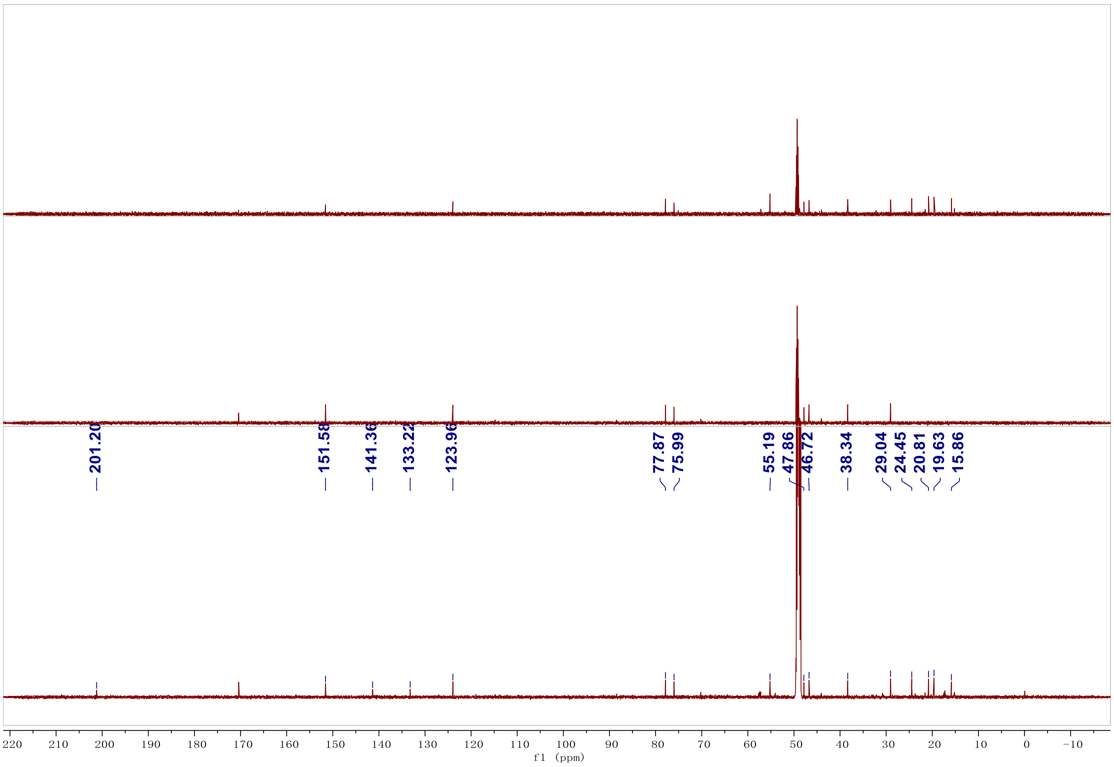

Figure S31. ^{13}C NMR spectrum of compound **3**. (150 MHz, methanol-d_4)

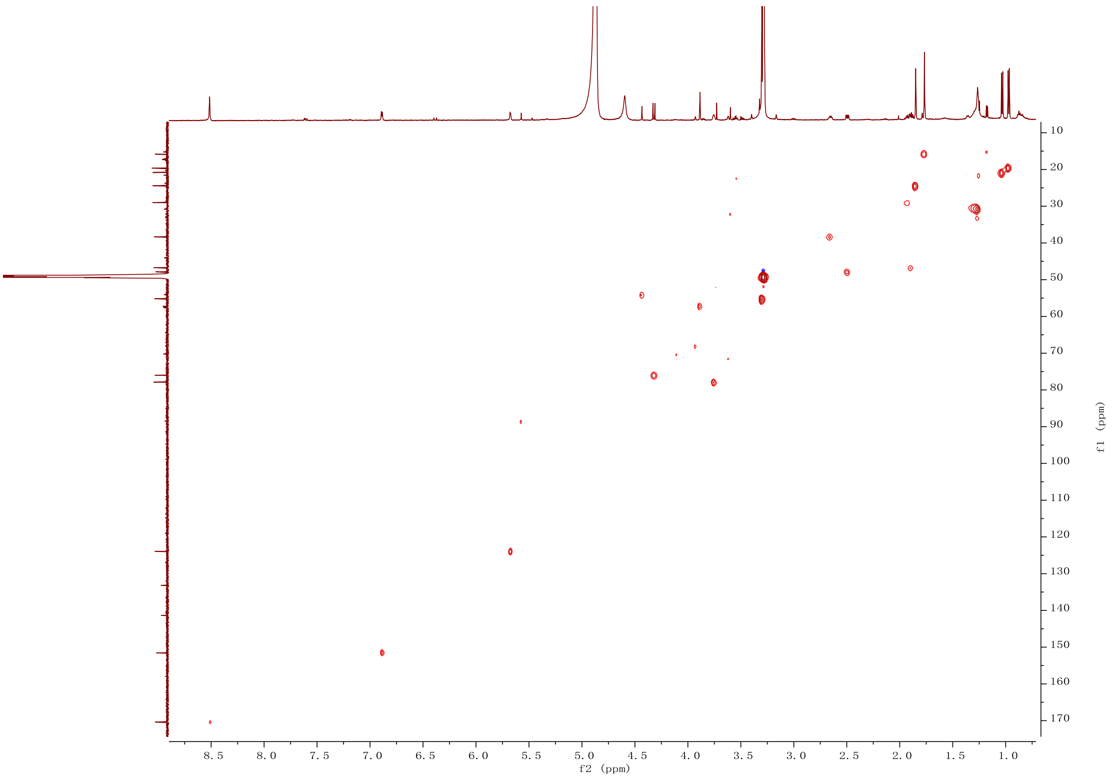

Figure S32. HSQC spectrum of compound **3**

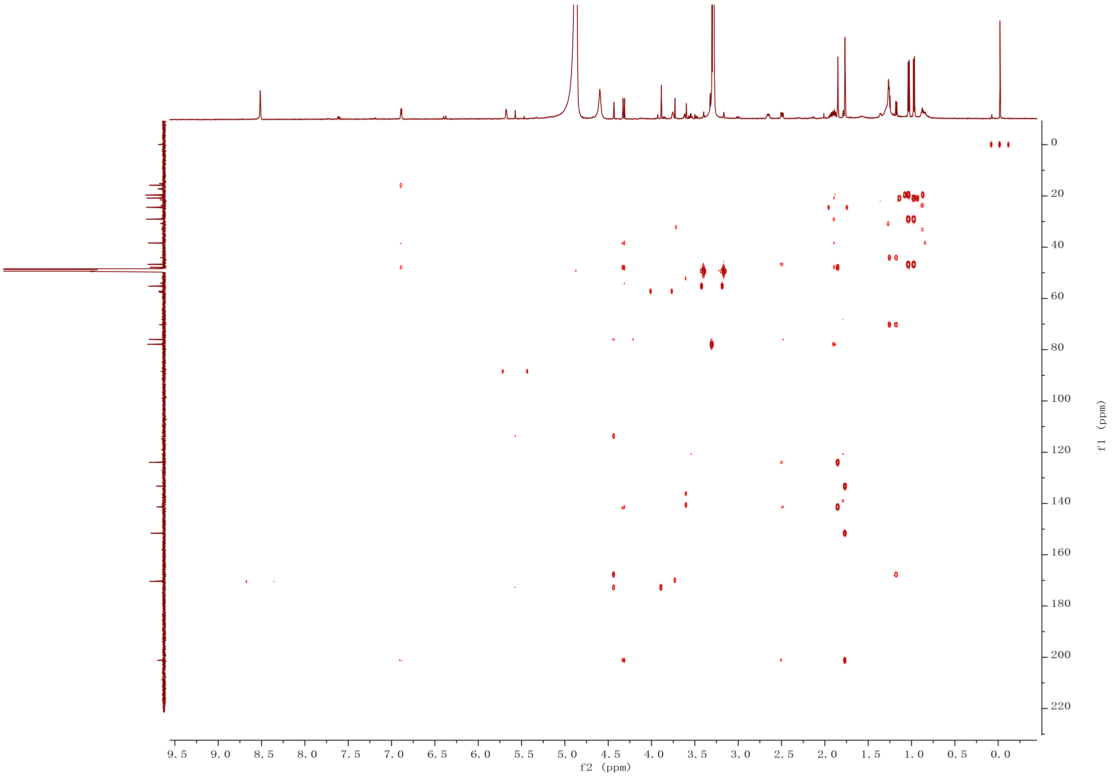

Figure S33. HMBC spectrum of compound **3**

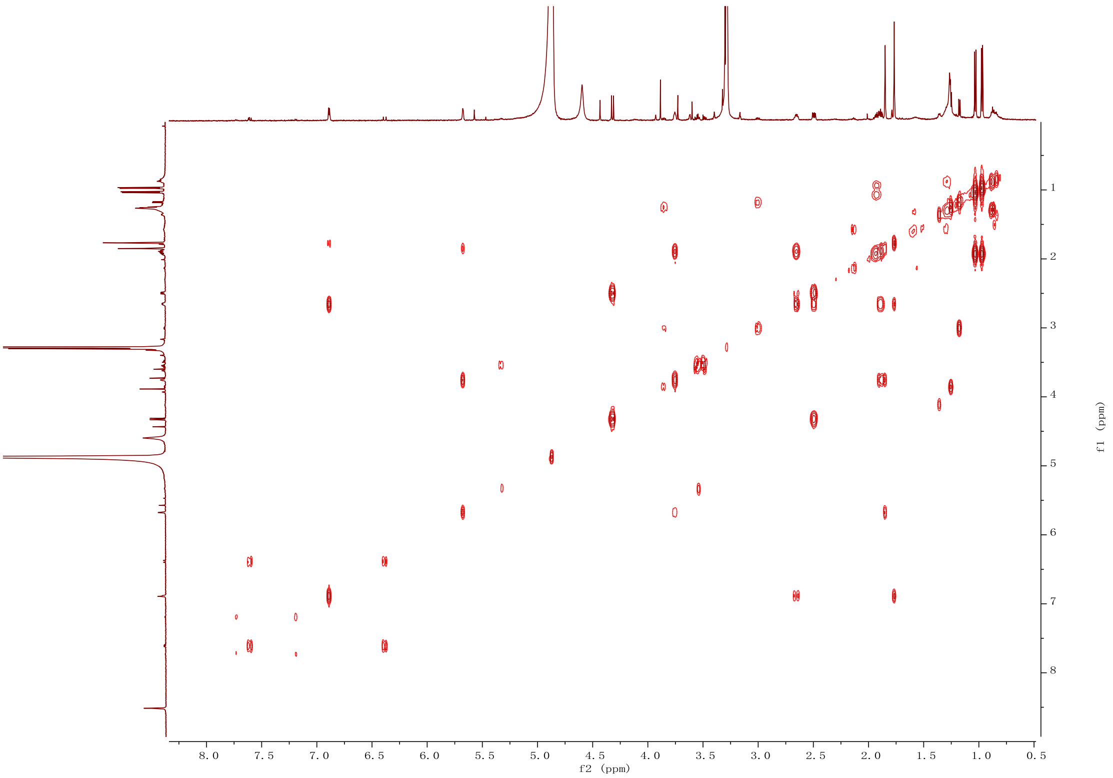

Figure S34. ^{1}H-^{1}H COSY spectrum of compound **3**

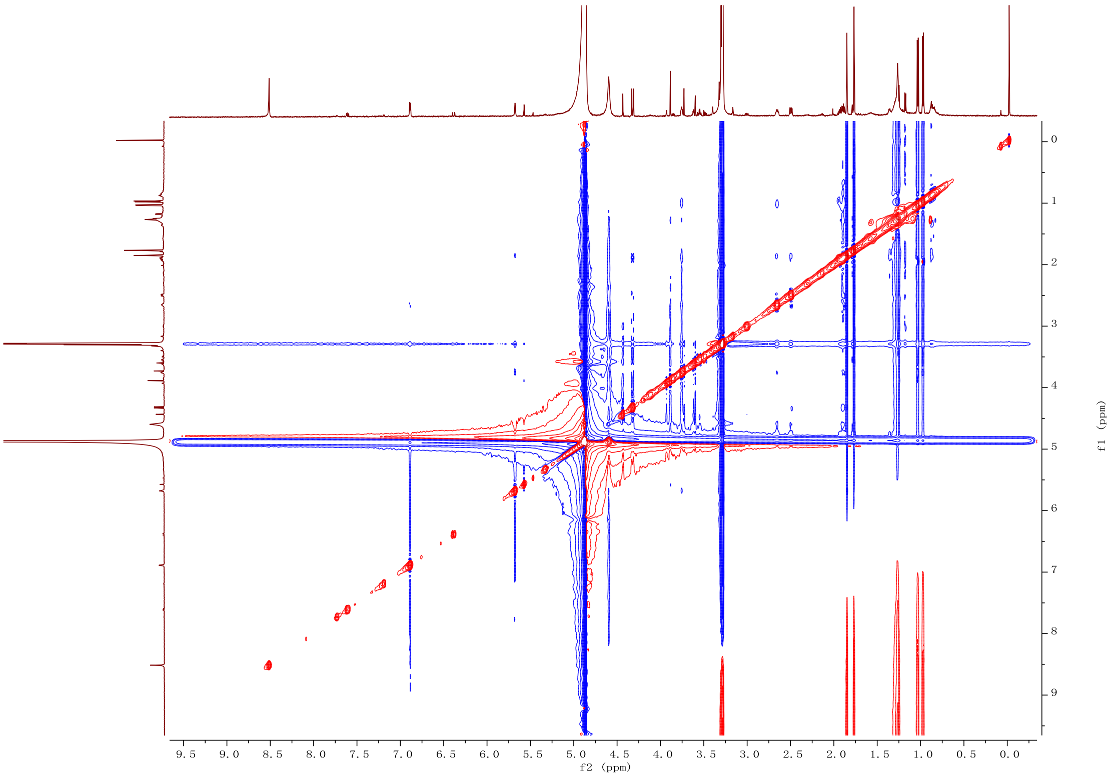

Figure S35. ROESY spectrum of compound **3**

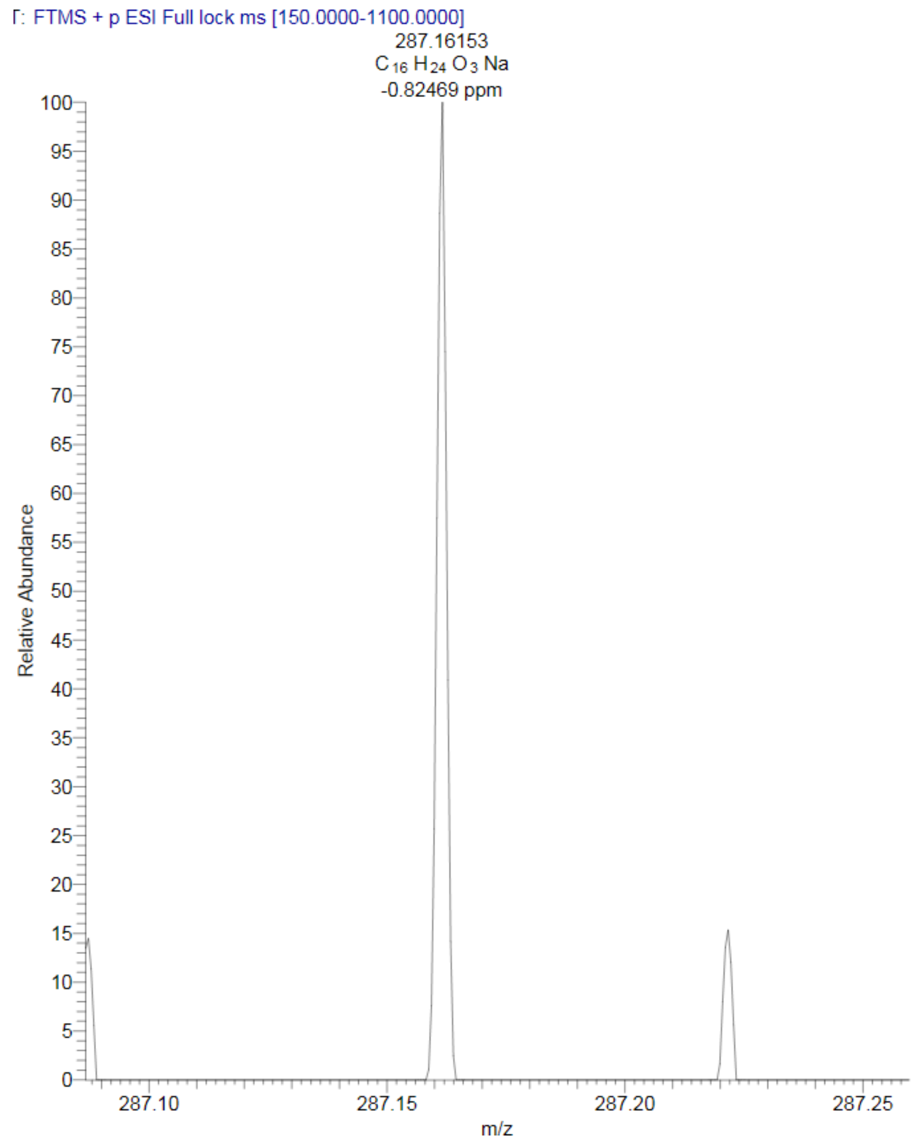

Figure S36. HRESIMS data of compound **3**

S3.4 NMR and HRESIMS spectra of albocinnamin D (4)

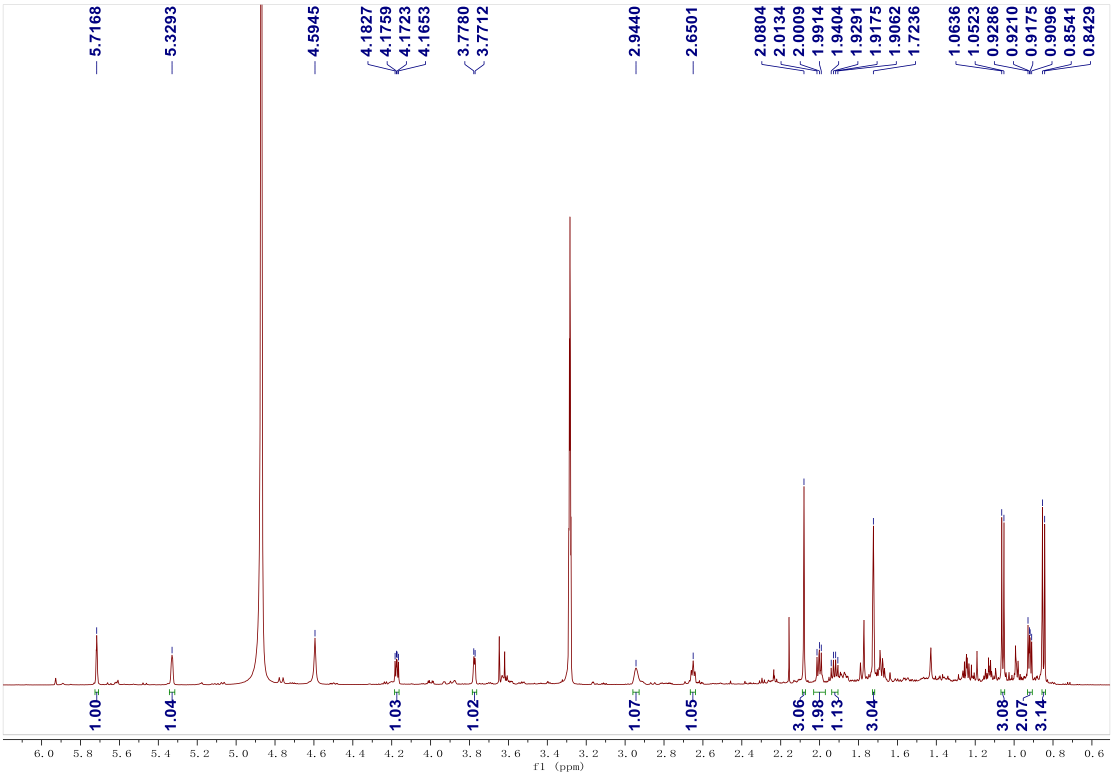

Figure S37. ^{1}H NMR spectrum of compound **4**. (600 MHz, methanol-*d*$_4$)

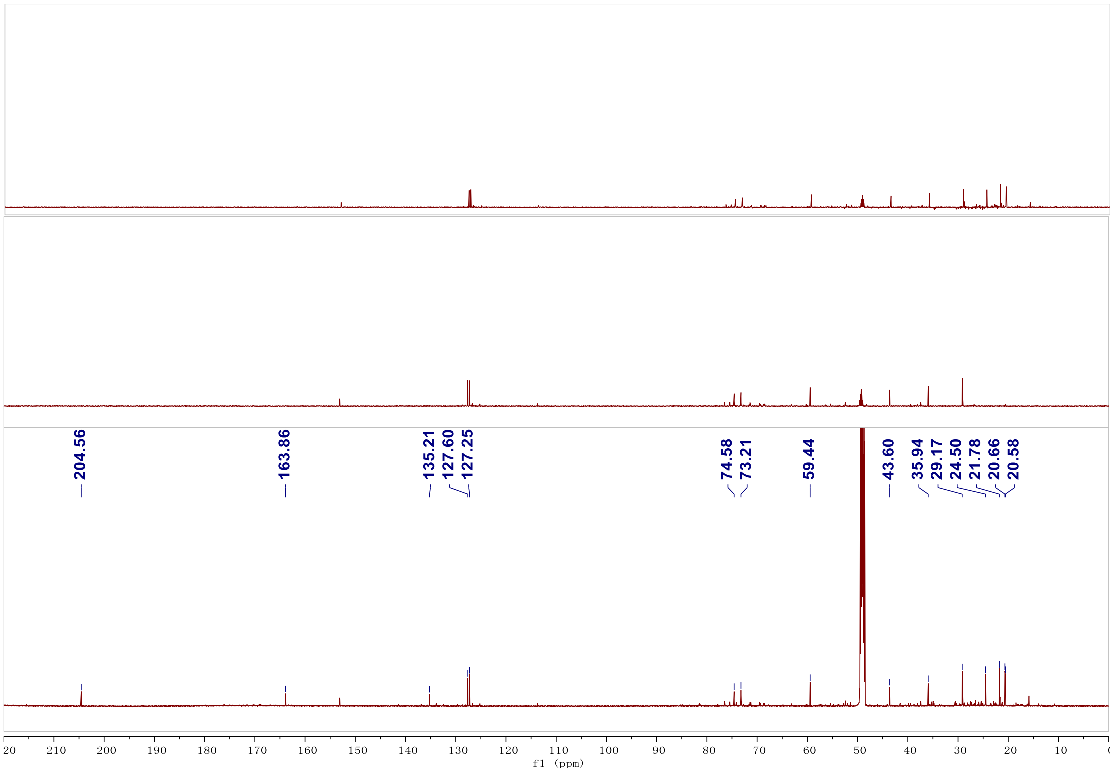

Figure S38. ^{13}C NMR spectrum of compound **4**. (150 MHz, methanol-*d*$_4$)

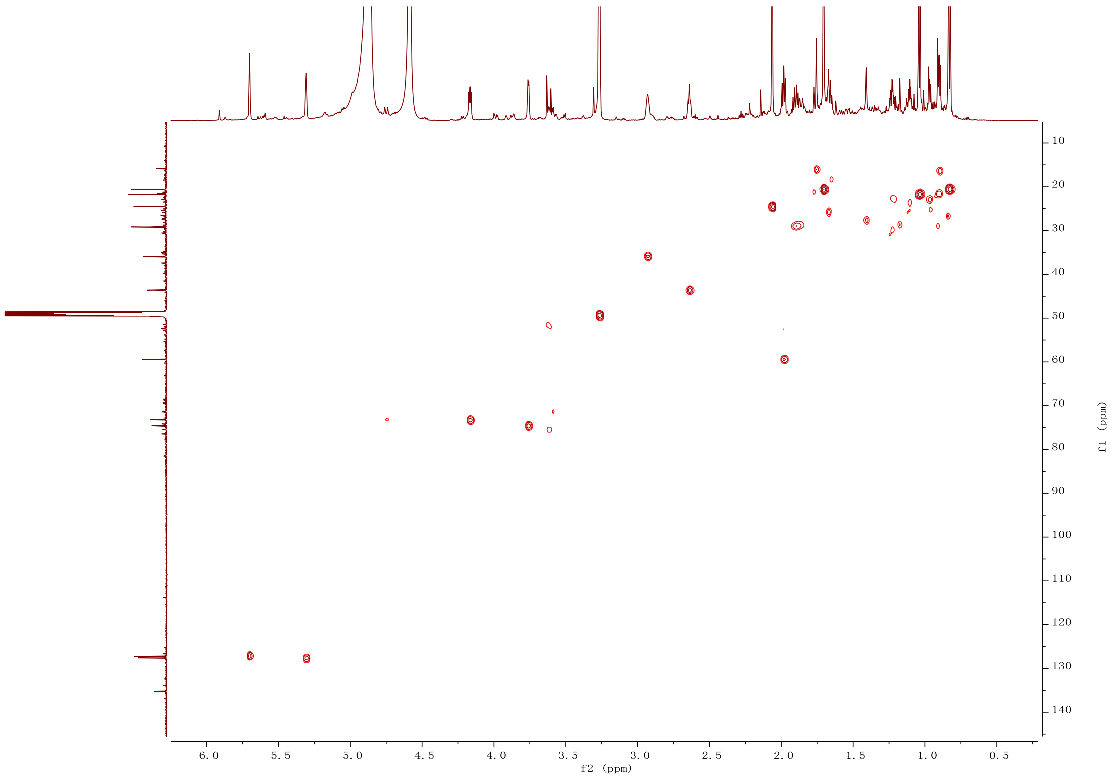

Figure S39. HSQC spectrum of compound **4**

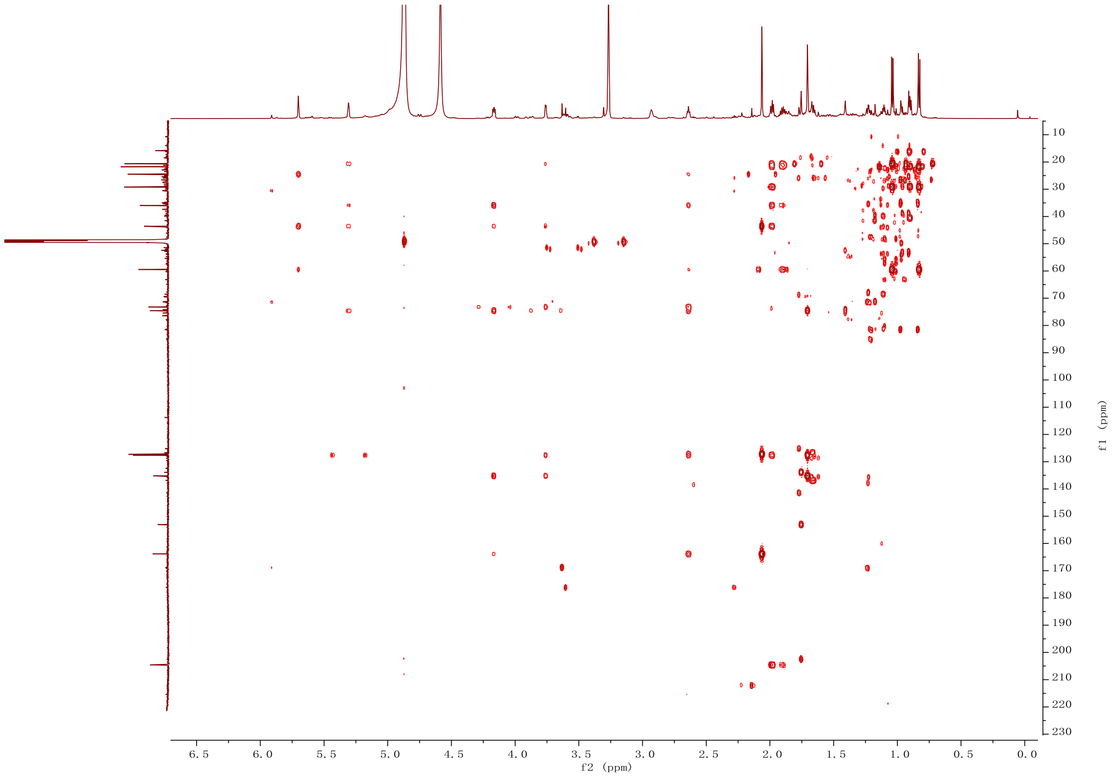

Figure S40. HMBC spectrum of compound **4**

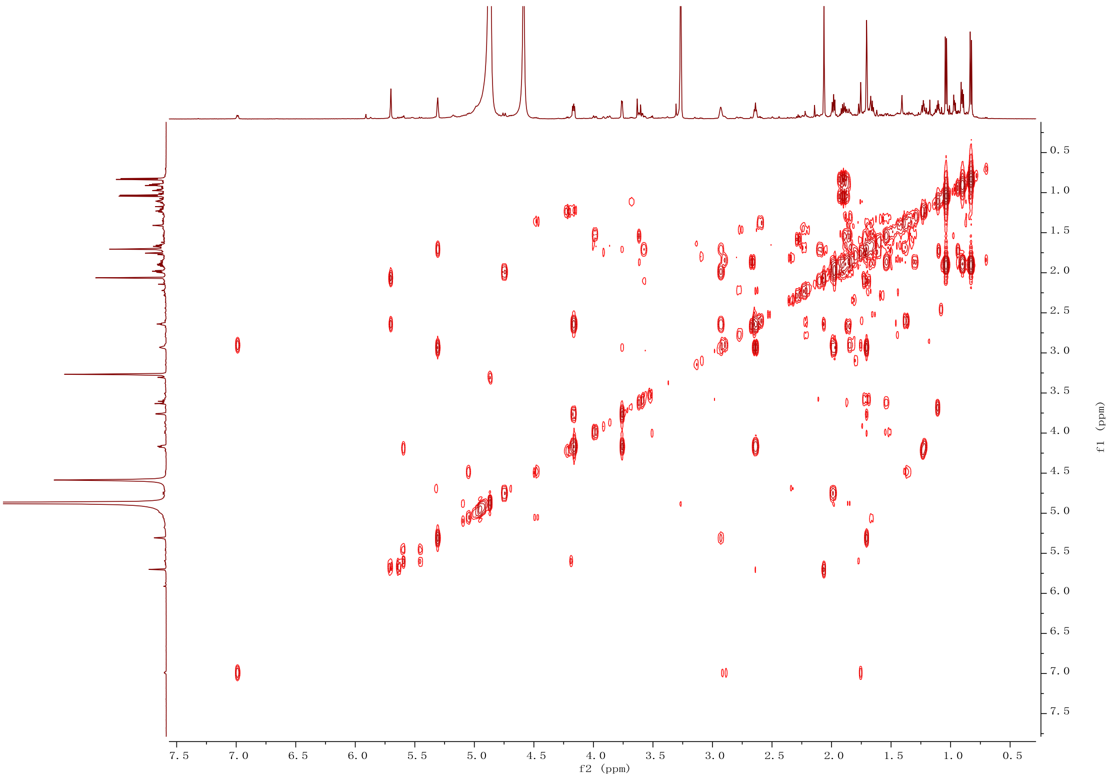

Figure S41. ^{1}H-^{1}H COSY spectrum of compound **4**

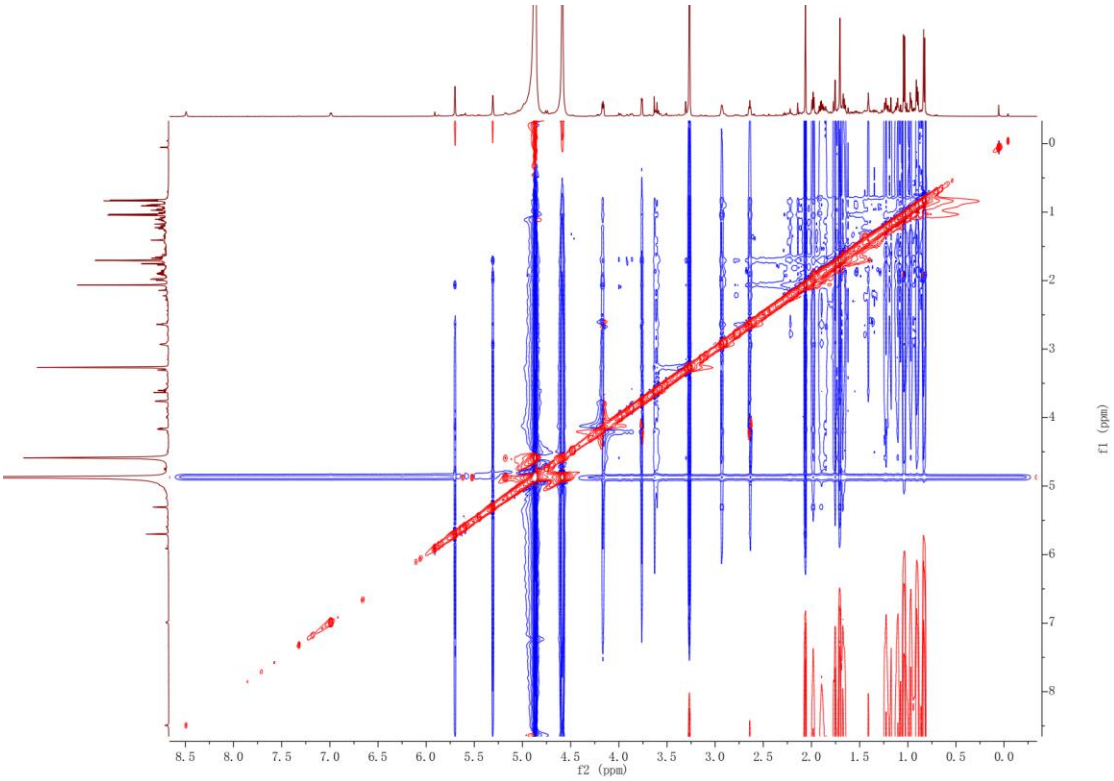

Figure S42. ROESY spectrum of compound **4**

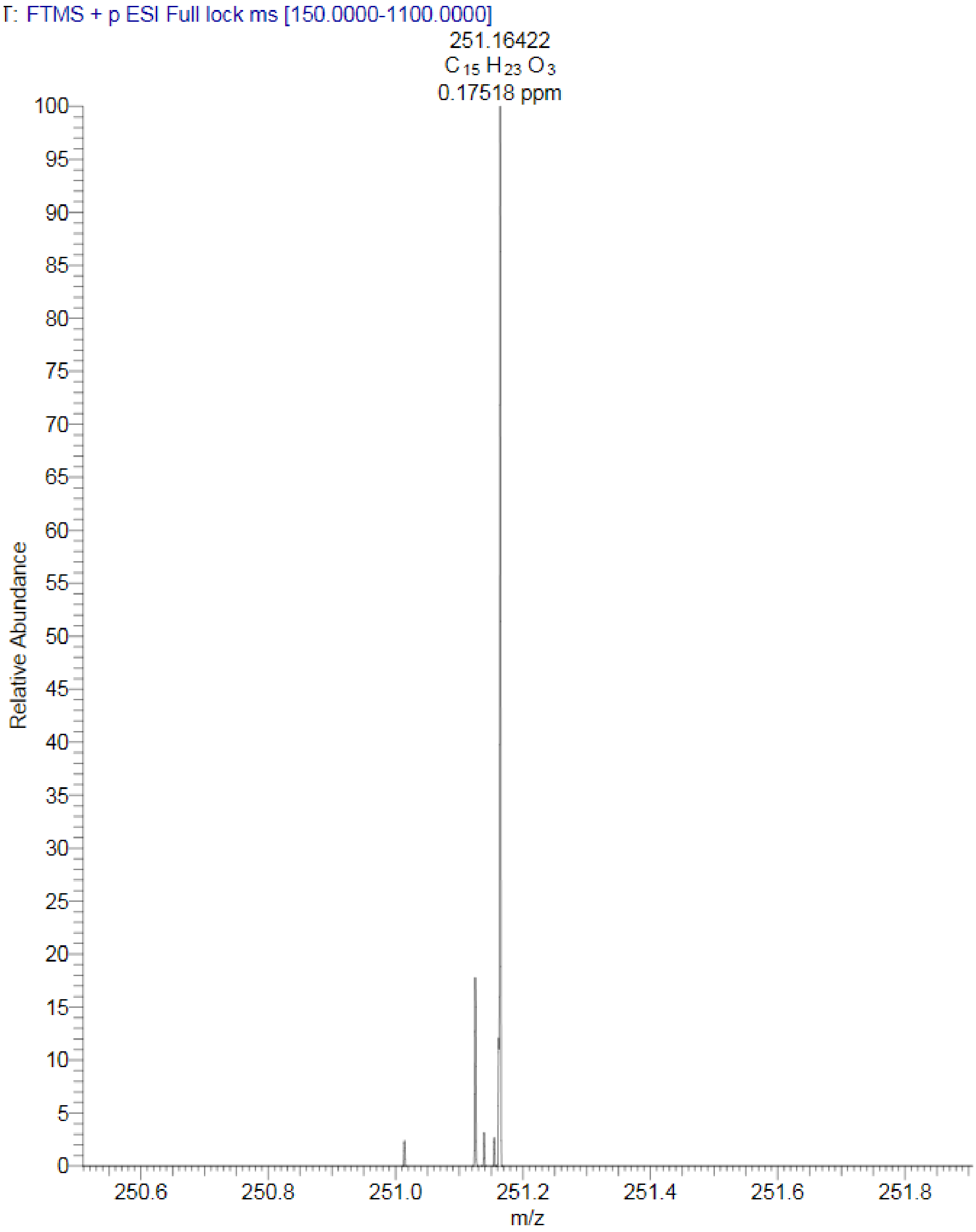

Figure S43. HRESIMS data of compound **4**

S3.5 NMR and HRESIMS spectra of albocinnamin E (5)

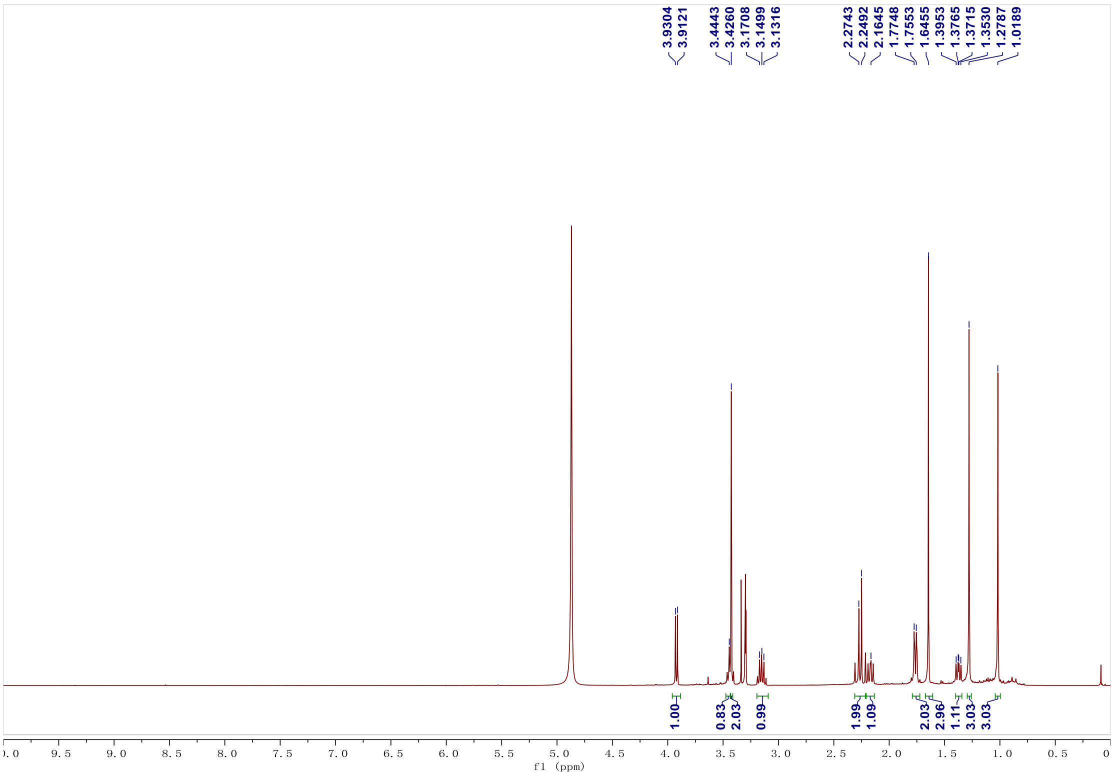

Figure S44. ^{1}H NMR spectrum of compound **5**. (600 MHz, methanol-d_4)

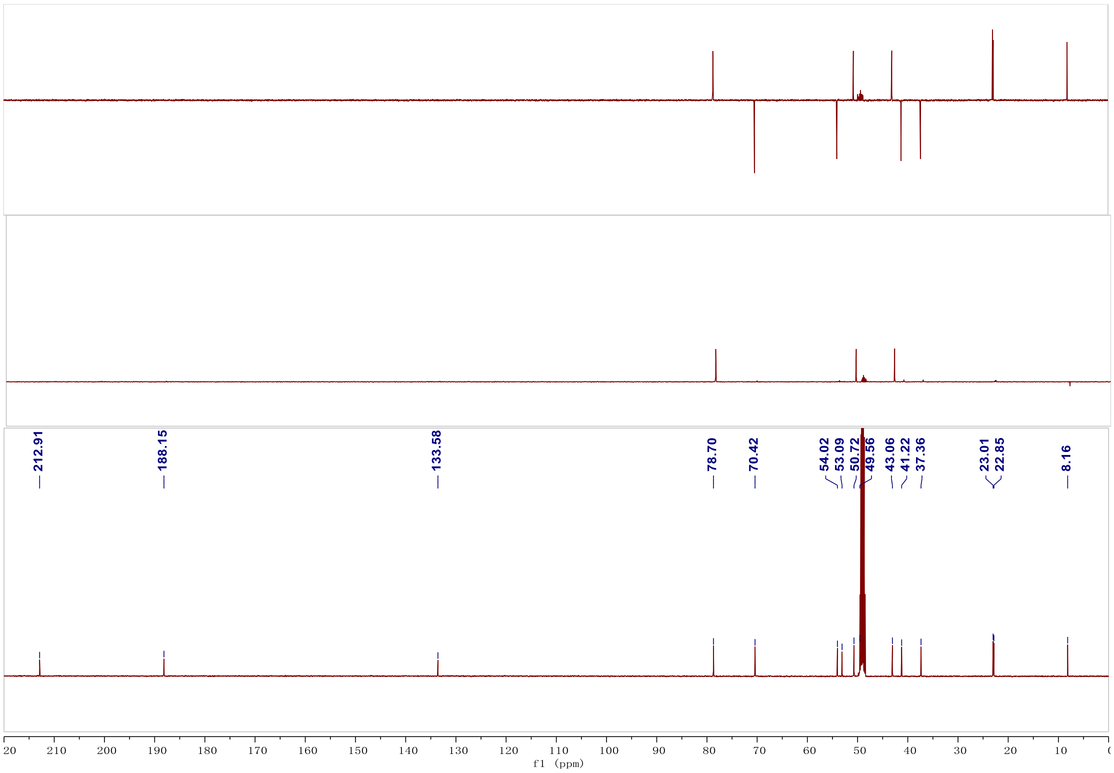

Figure S45. ^{13}C NMR spectrum of compound **5**. (150 MHz, methanol-d_4)

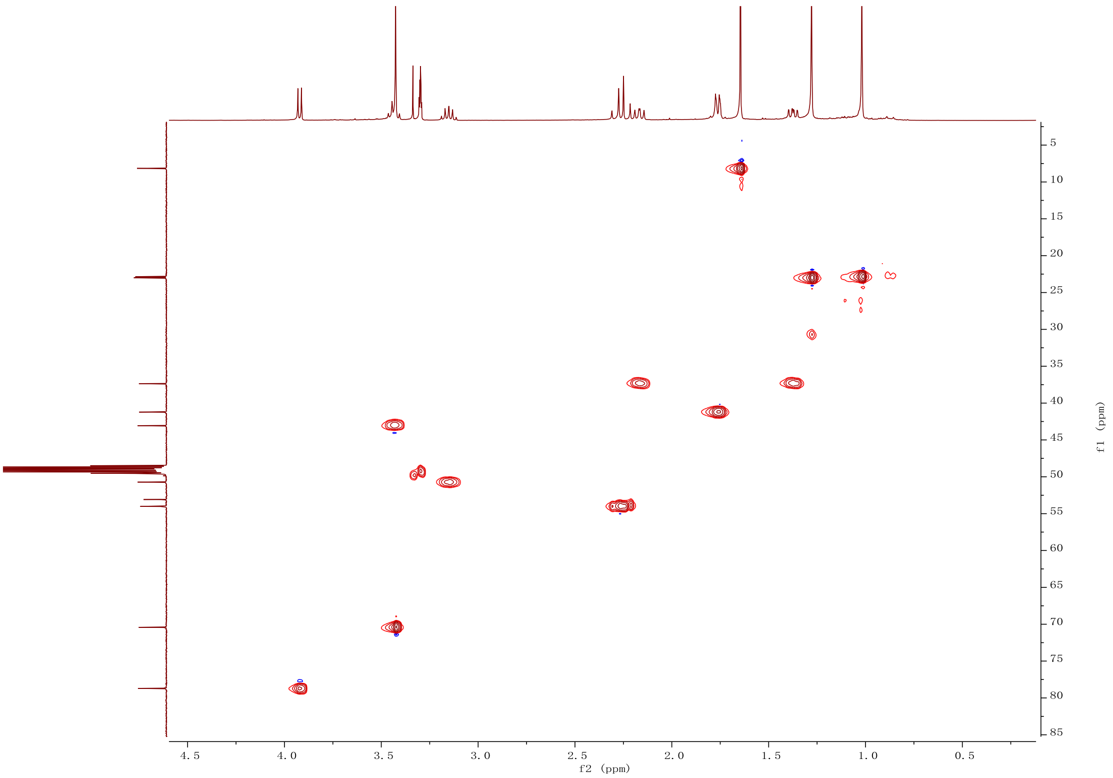

Figure S46. HSQC spectrum of compound **5**

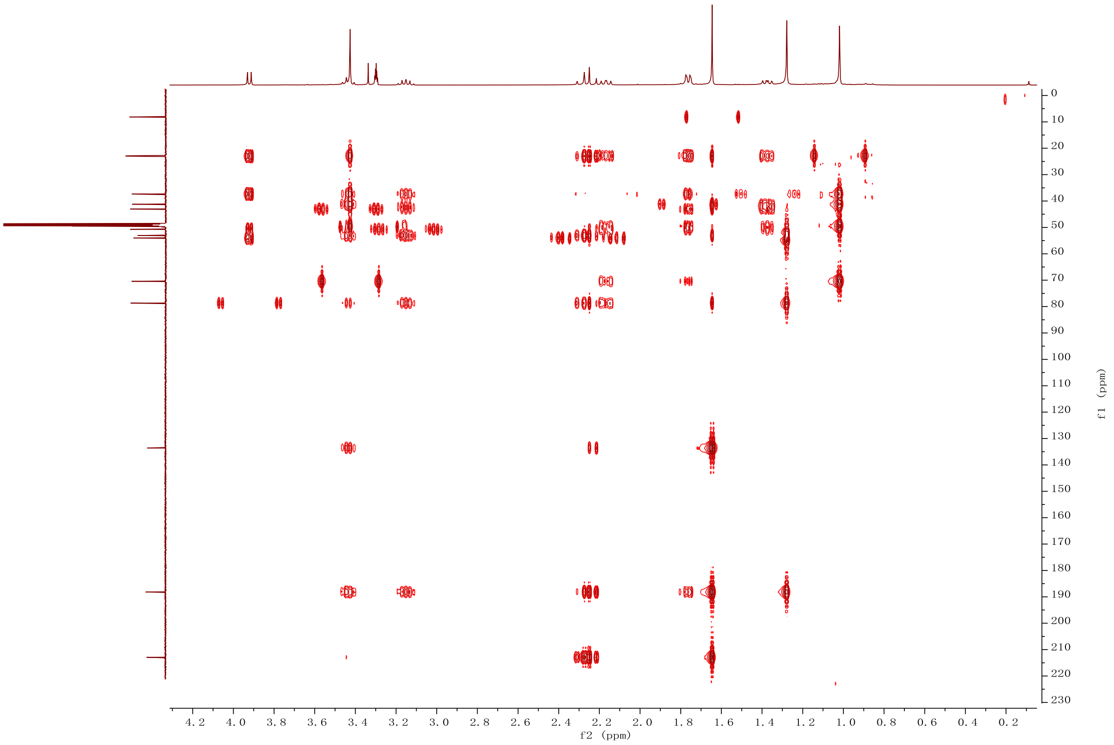

Figure S47. HMBC spectrum of compound **5**

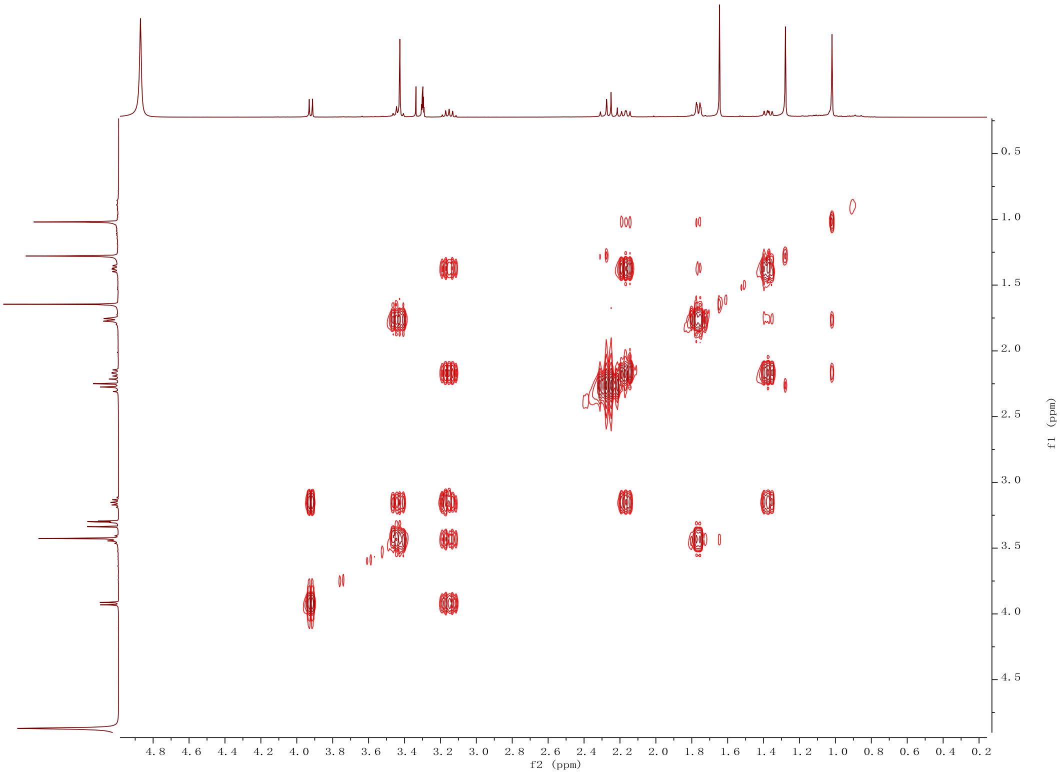

Figure S48. ^1H-^1H COSY spectrum of compound **5**

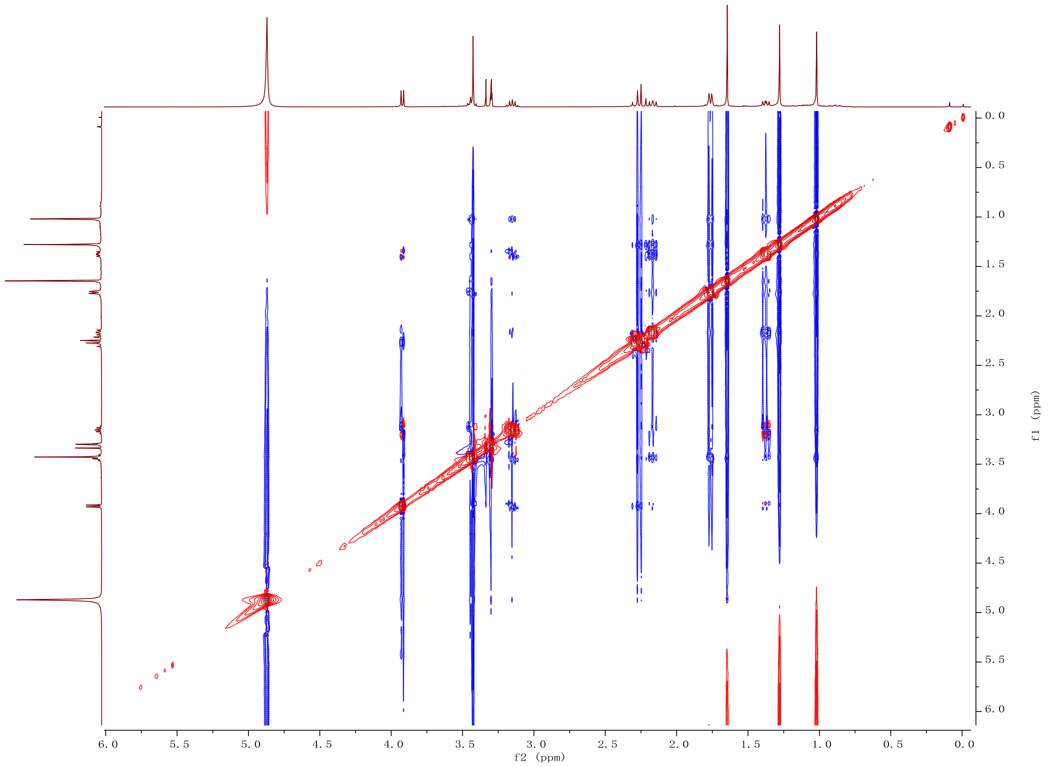

Figure S49. ROESY spectrum of compound **5**

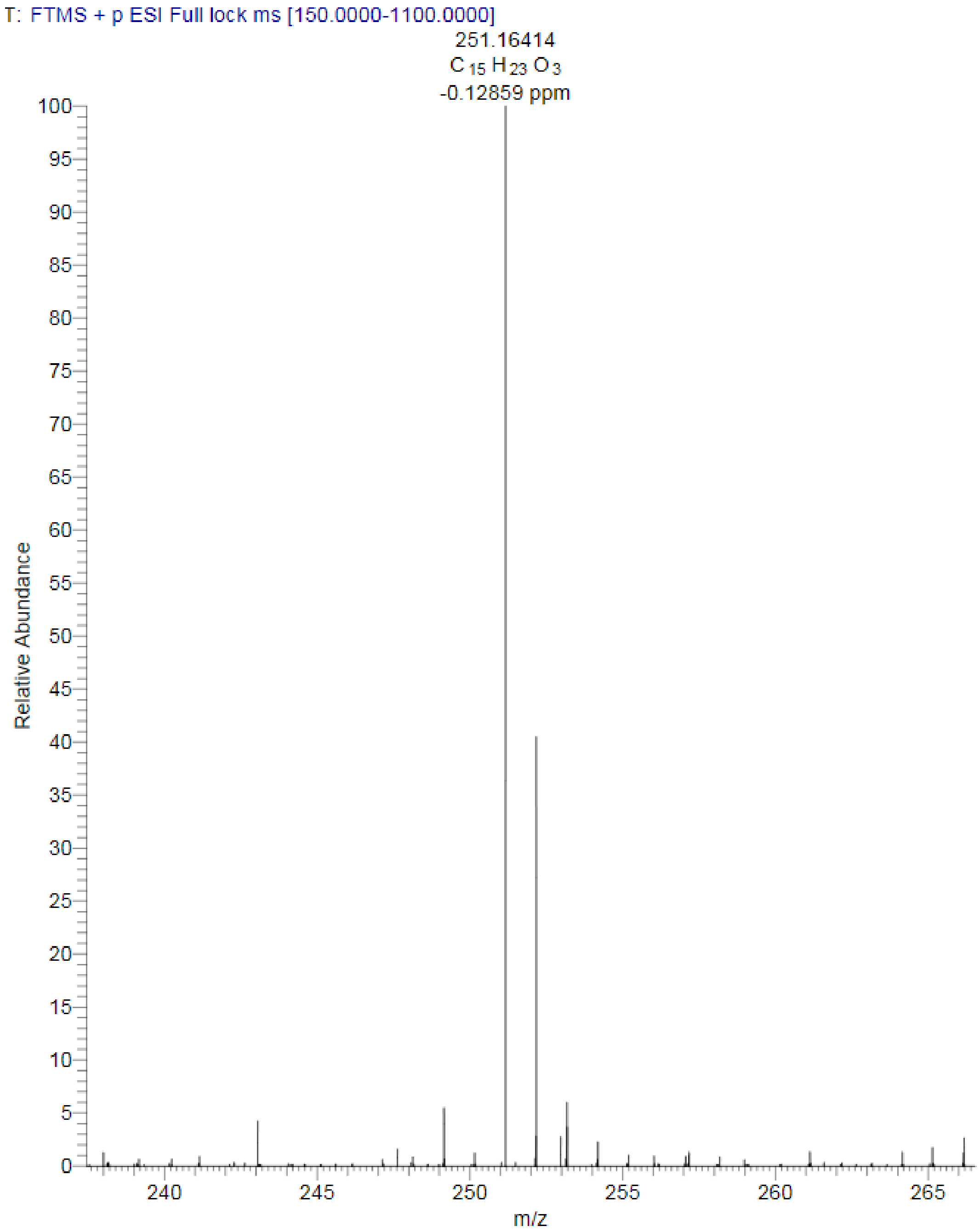

Figure S50. HRESIMS data of compound **5**

S3.6 NMR and HRESIMS spectra of albocinnamin F (6)

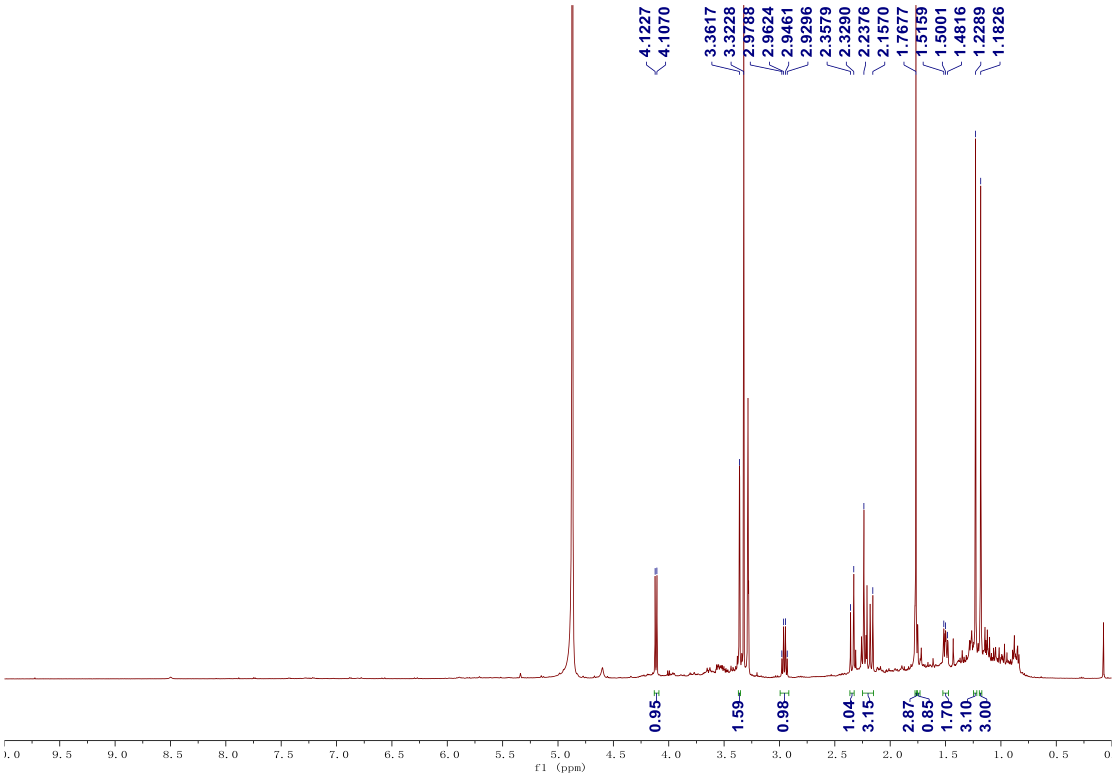

Figure S51. ^{1}H NMR spectrum of compound **6**. (600 MHz, DMSO-d_6)

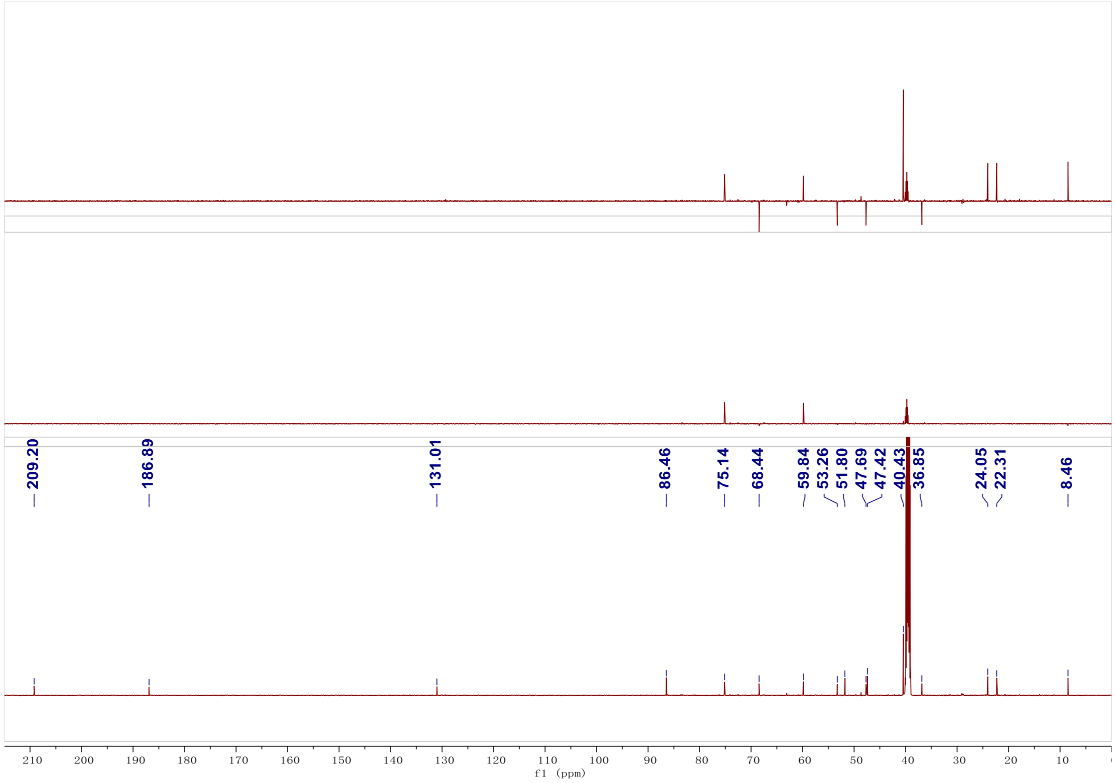

Figure S52. ^{13}C NMR spectrum of compound **6**. (150 MHz, DMSO-d_6)

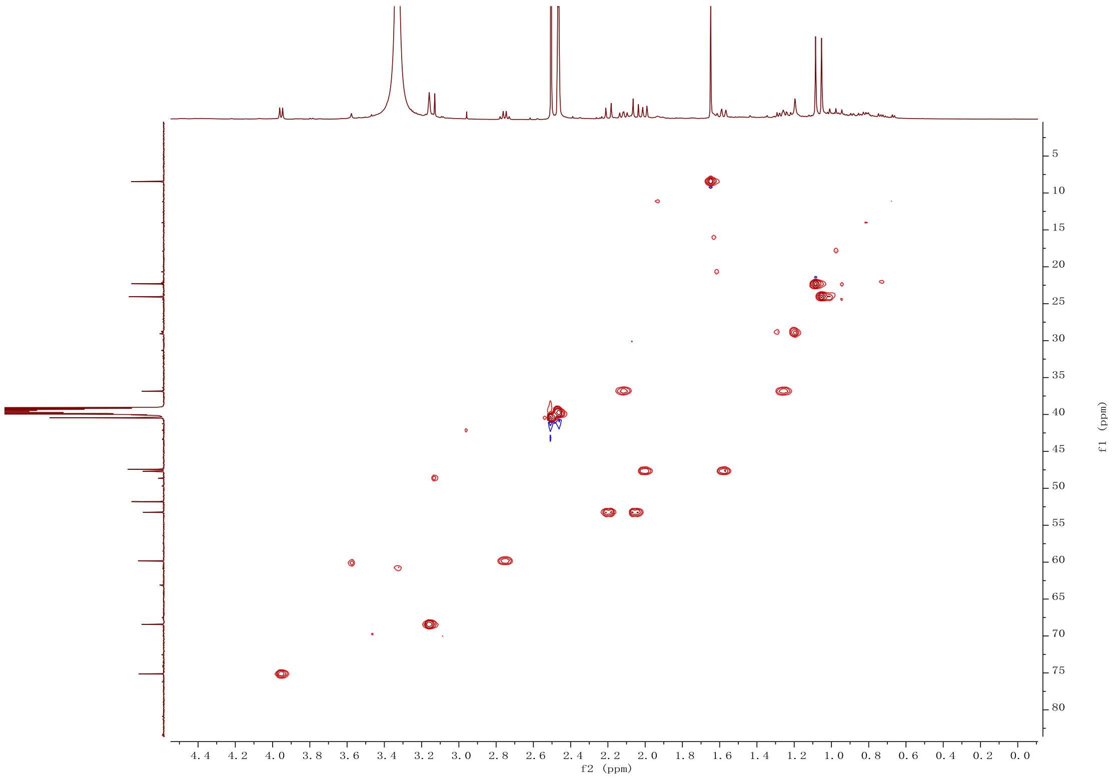

Figure S53. HSQC spectrum of compound **6**

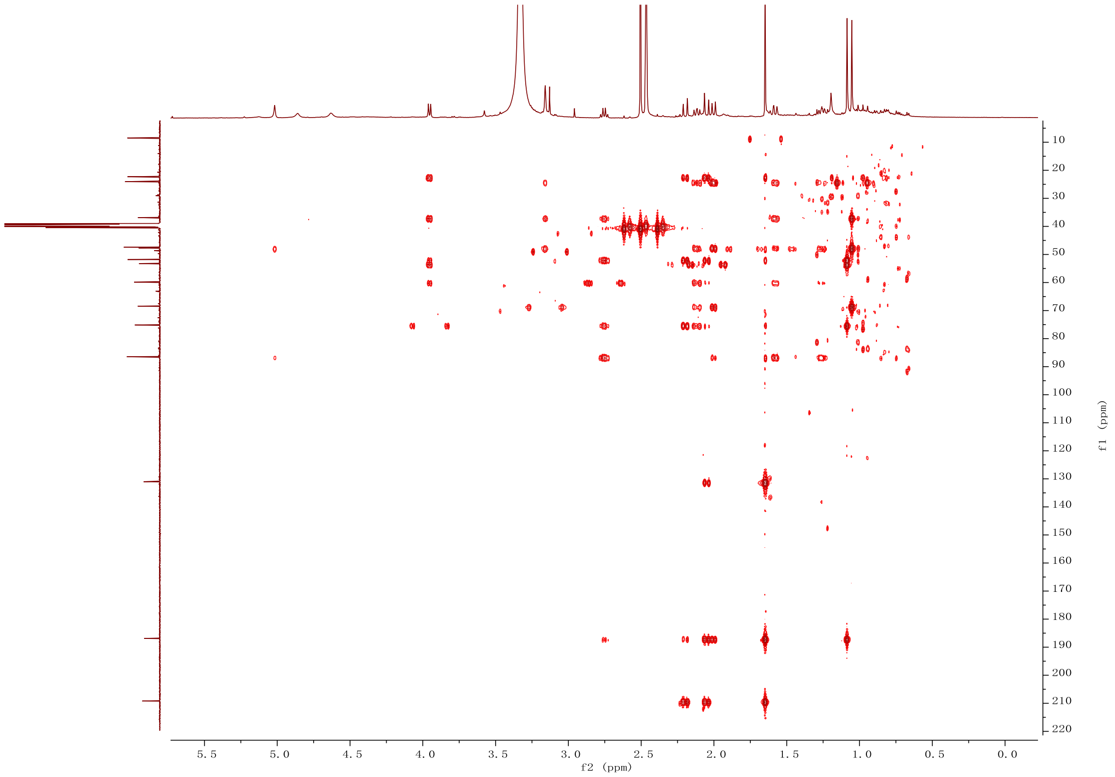

Figure S54. HMBC spectrum of compound **6**

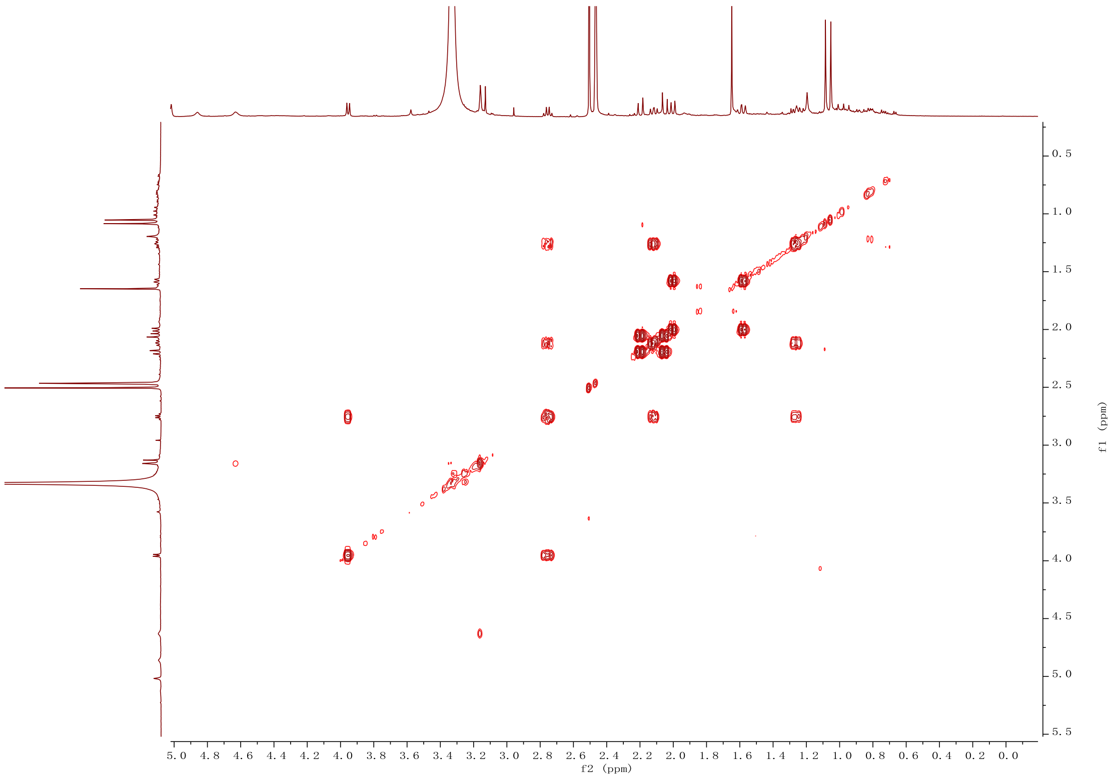

Figure S55. ^{1}H-^{1}H COSY spectrum of compound **6**

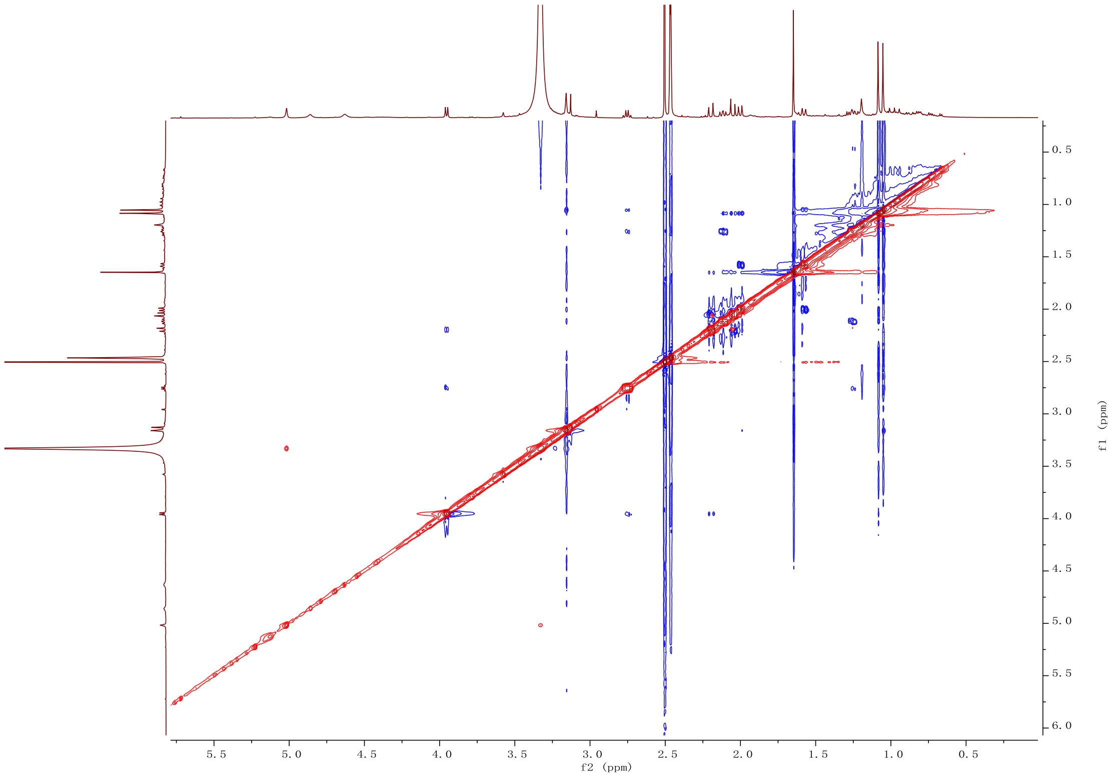

Figure S56. ROESY spectrum of compound **6**

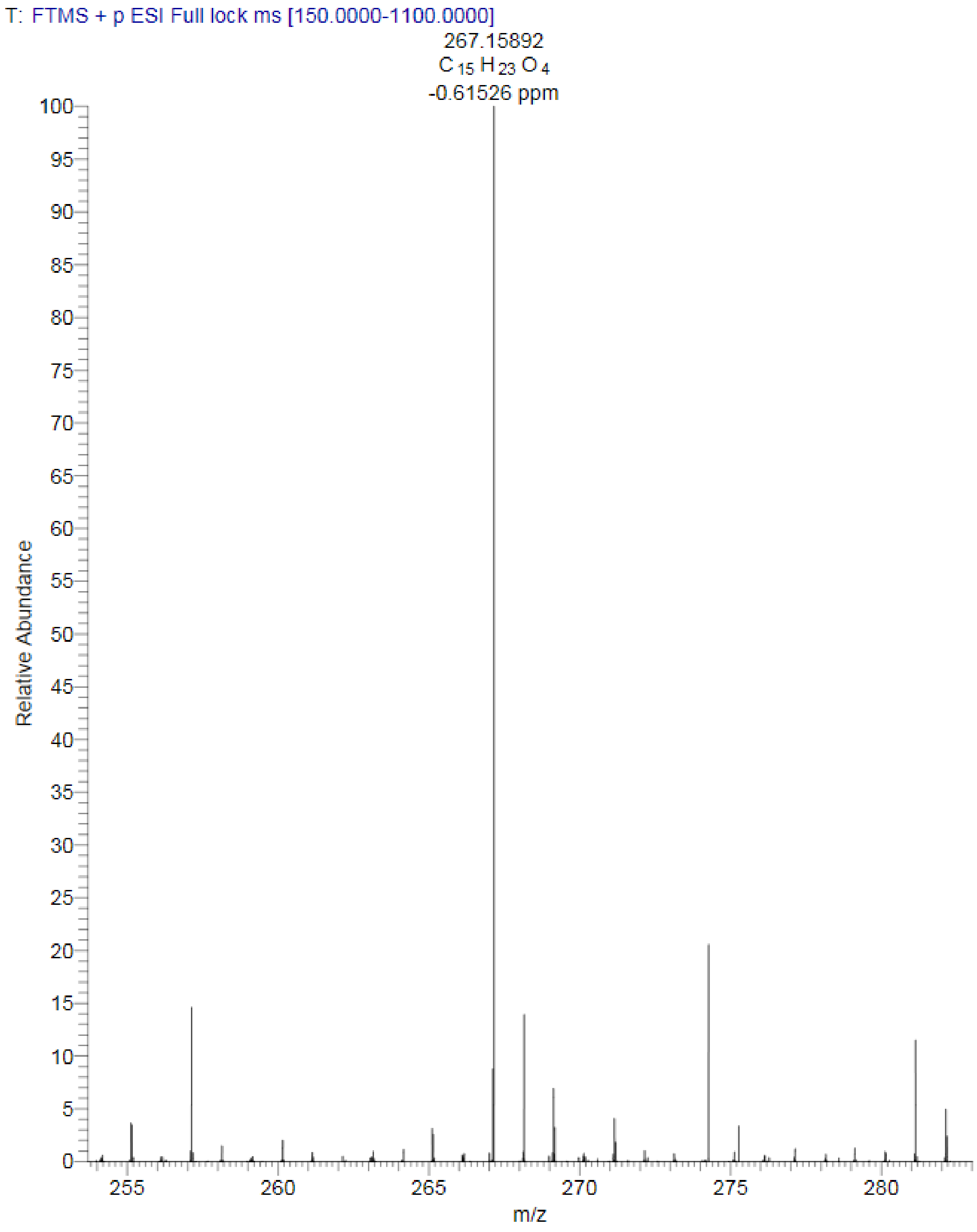

Figure S57. HRESIMS data of compound **6**

S3.7 NMR and HRESIMS spectra of albocinnamin G (7)

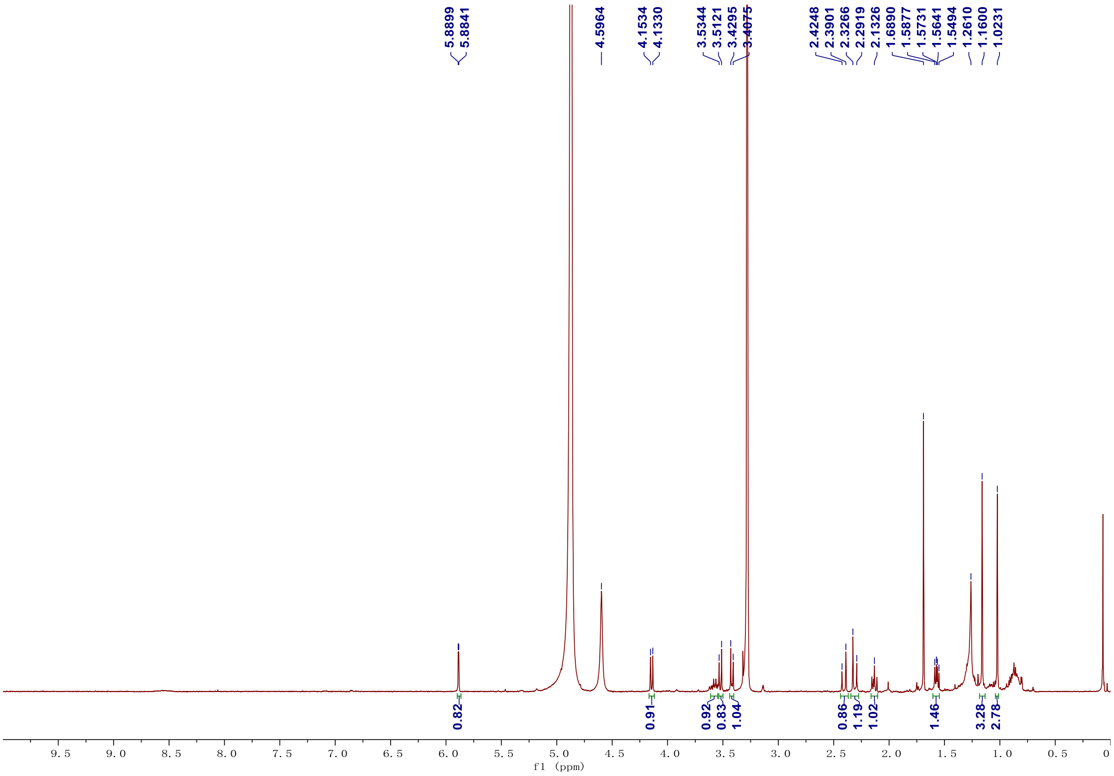

Figure S58. ^{1}H NMR spectrum of compound **7**. (600 MHz, methanol-d_4)

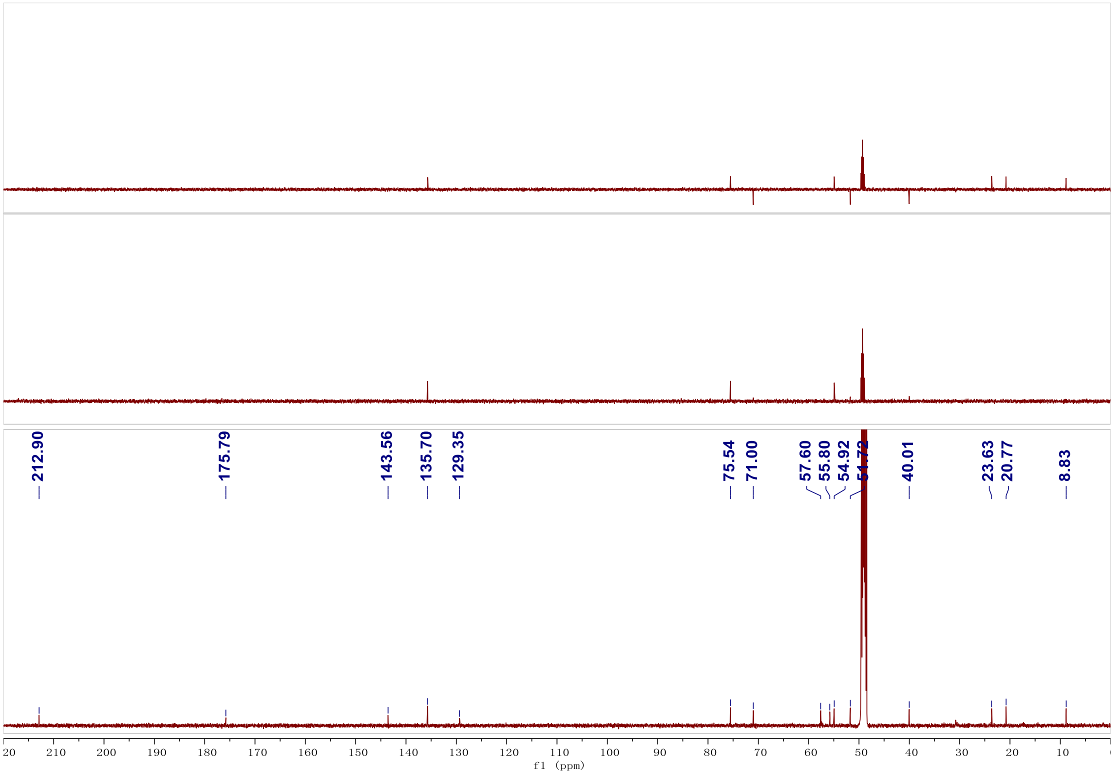

Figure S59. ^{13}C NMR spectrum of compound **7**. (150 MHz, methanol-d_4)

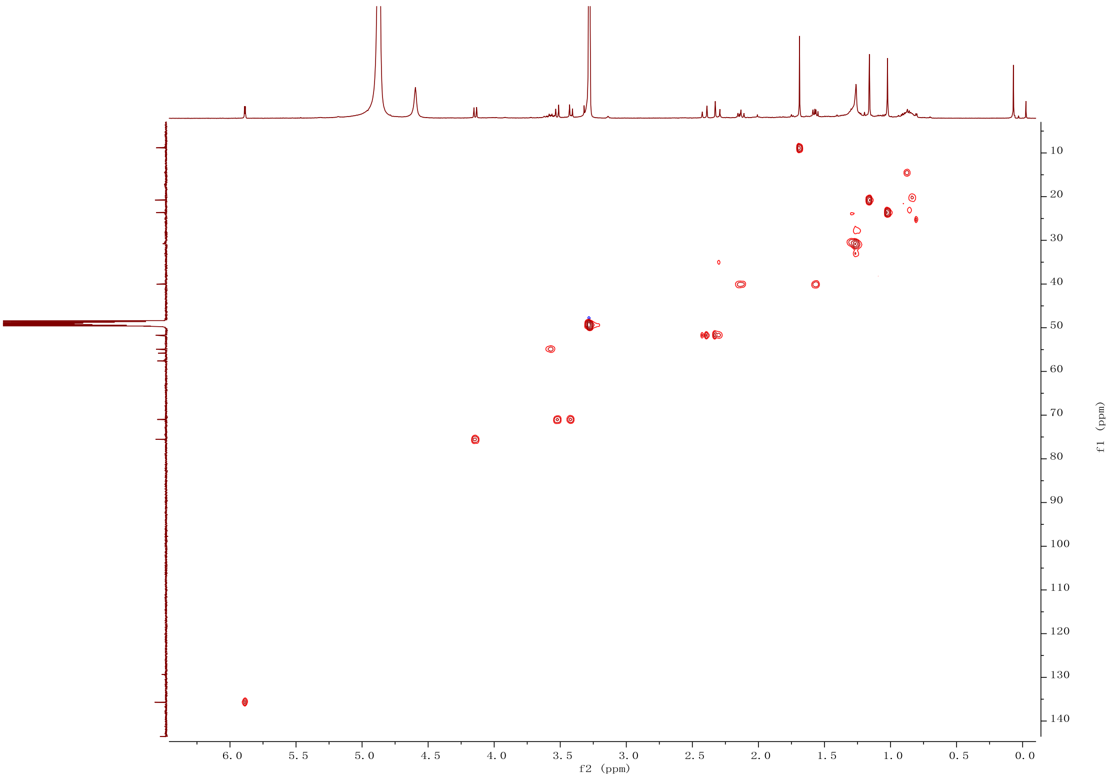

Figure S60. HSQC spectrum of compound **7**

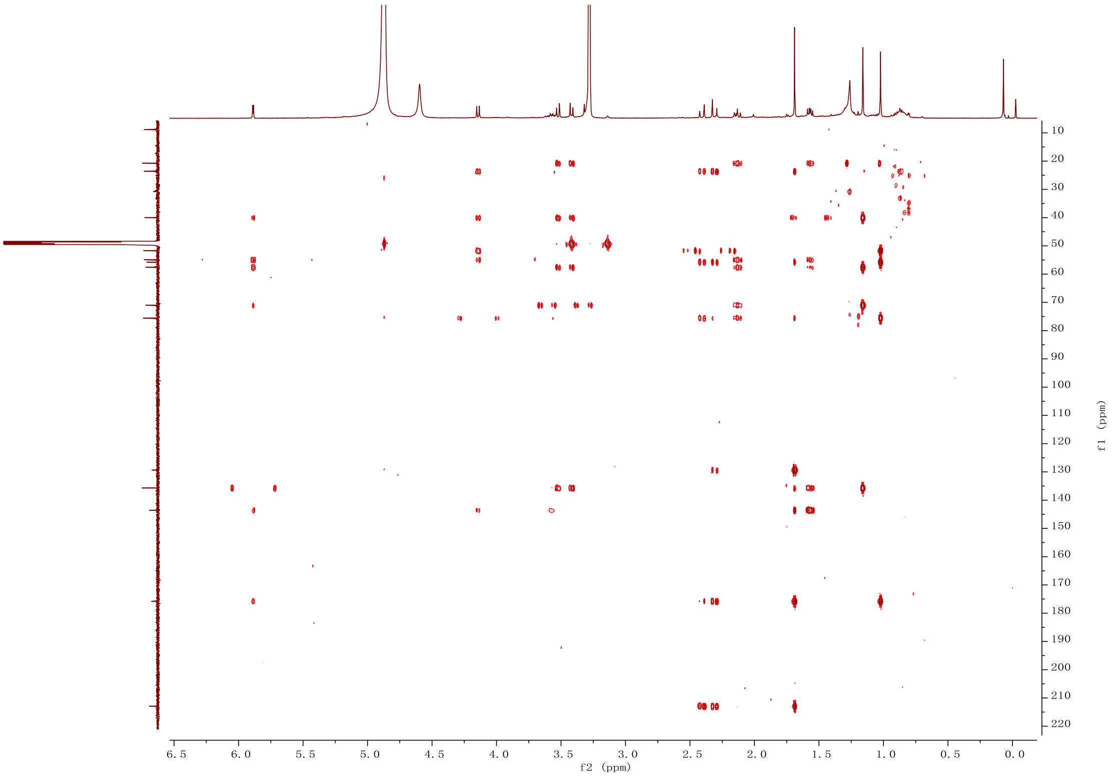

Figure S61. HMBC spectrum of compound **7**

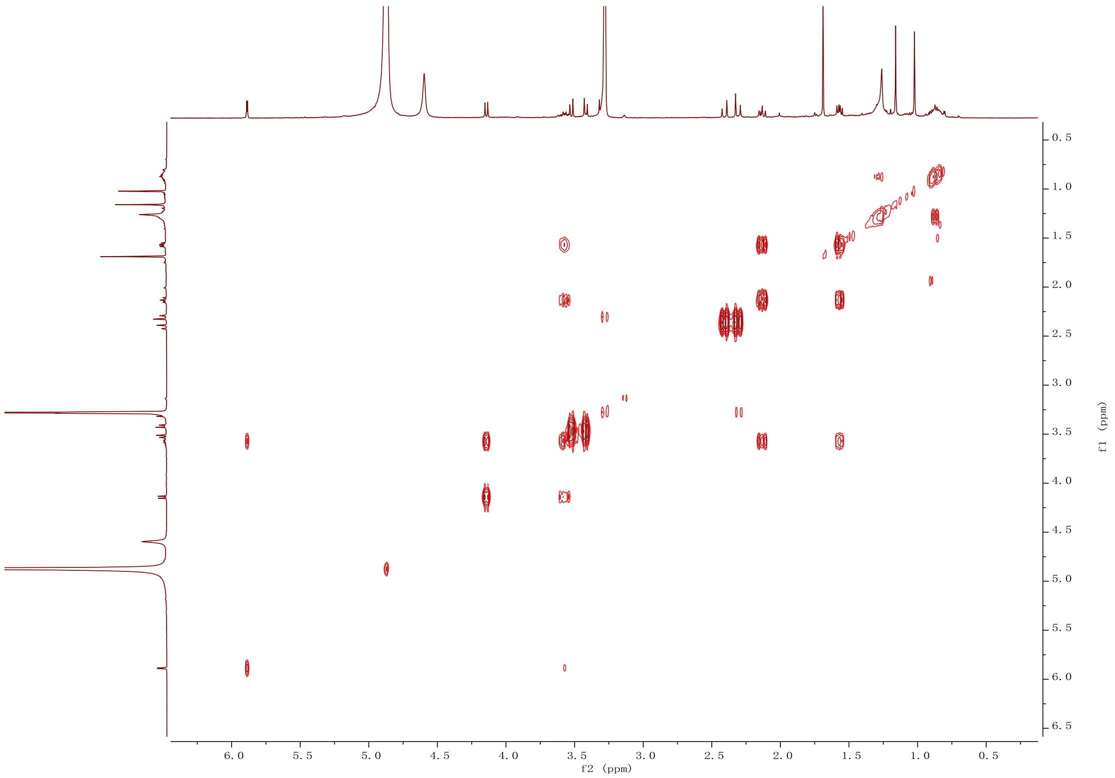

Figure S62. ^{1}H-^{1}H COSY spectrum of compound **7**

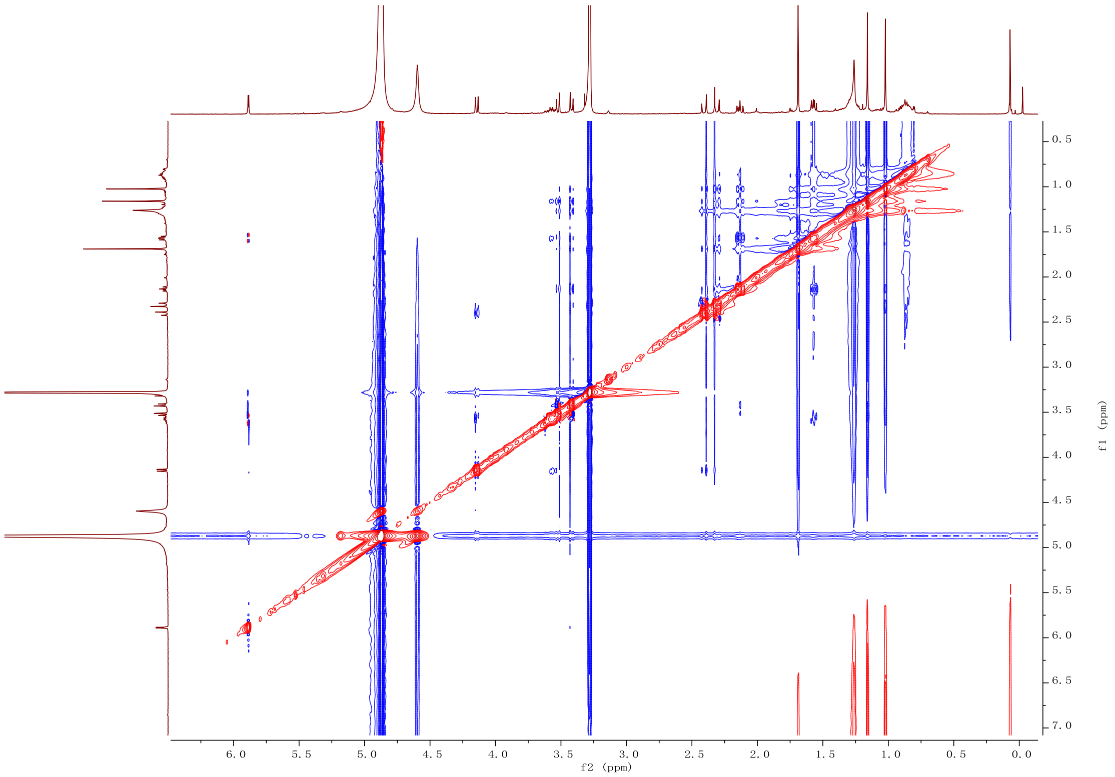

Figure S63. ROESY spectrum of compound **7**

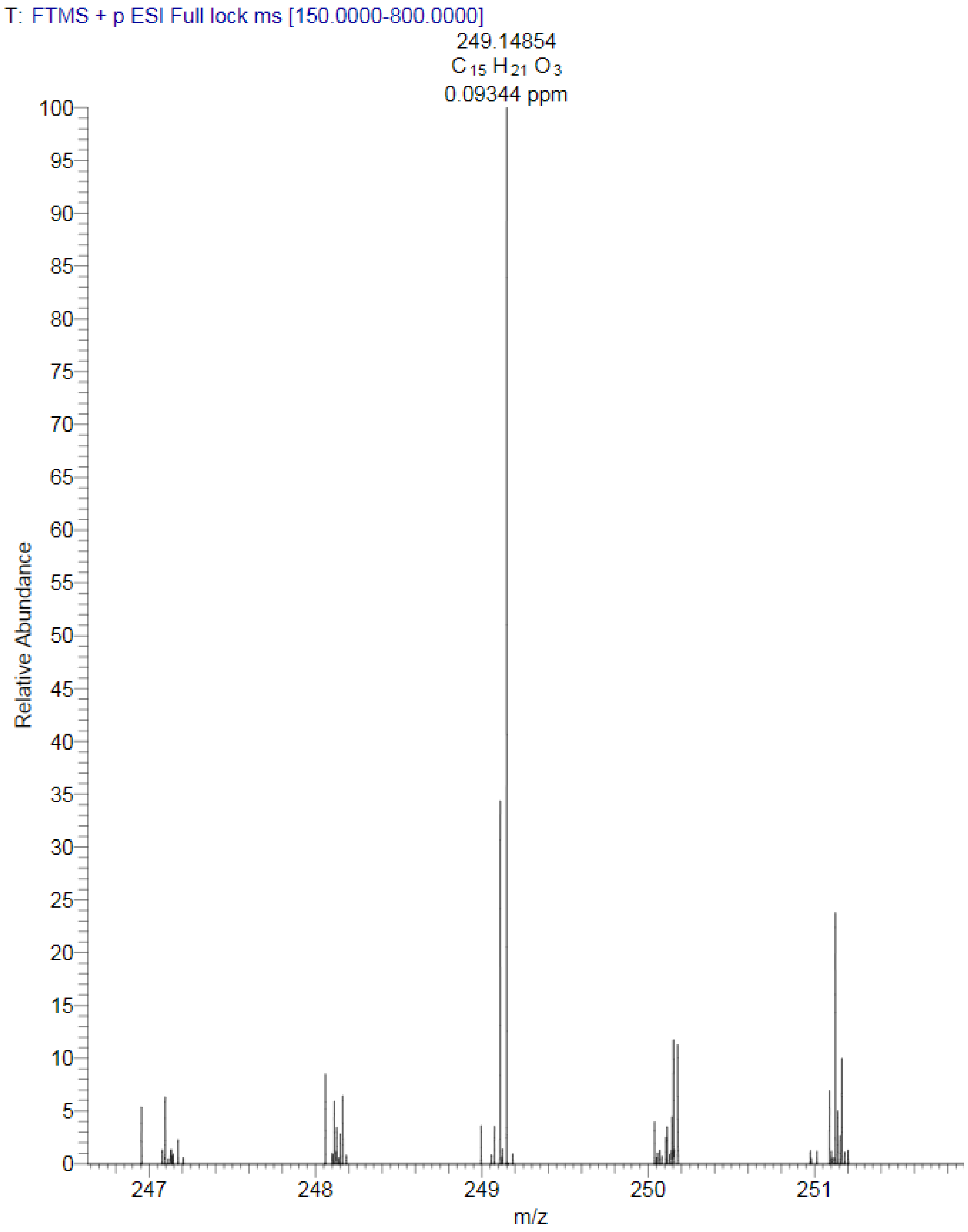

Figure S64. HRESIMS data of compound **7**

S3.8 NMR and HRESIMS spectra of albocinnamin H (8)

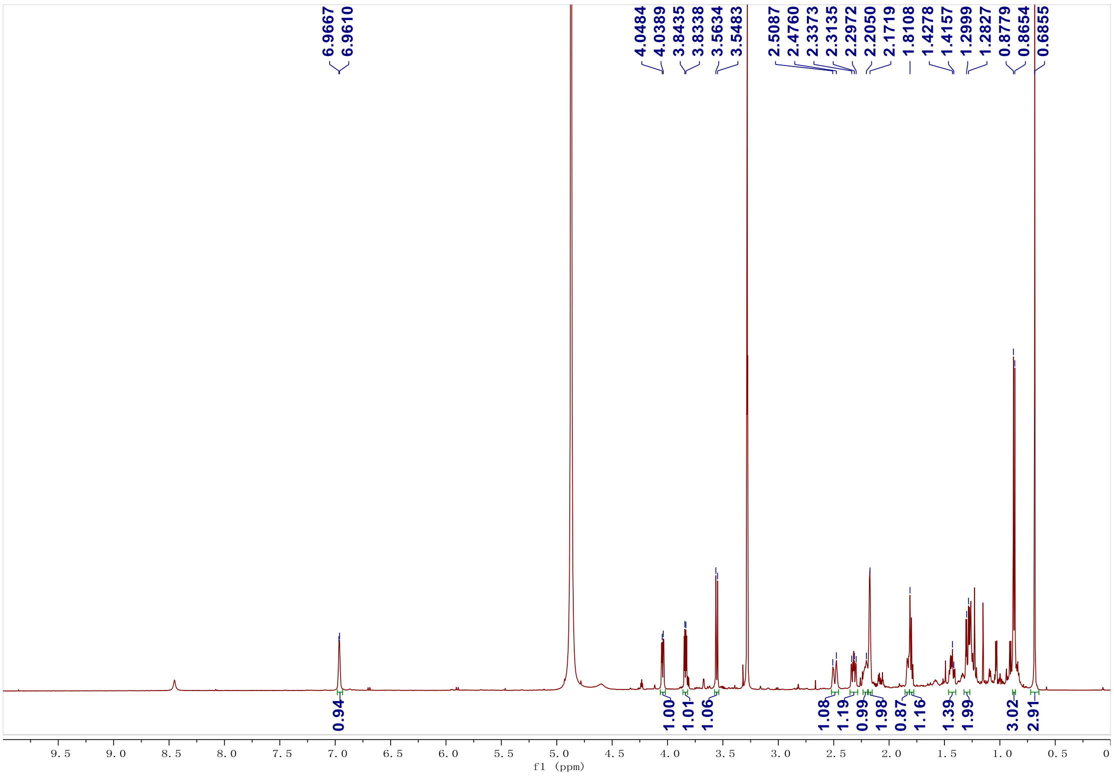

Figure S65. ^{1}H NMR spectrum of compound **8**. (600 MHz, methanol-d_4)

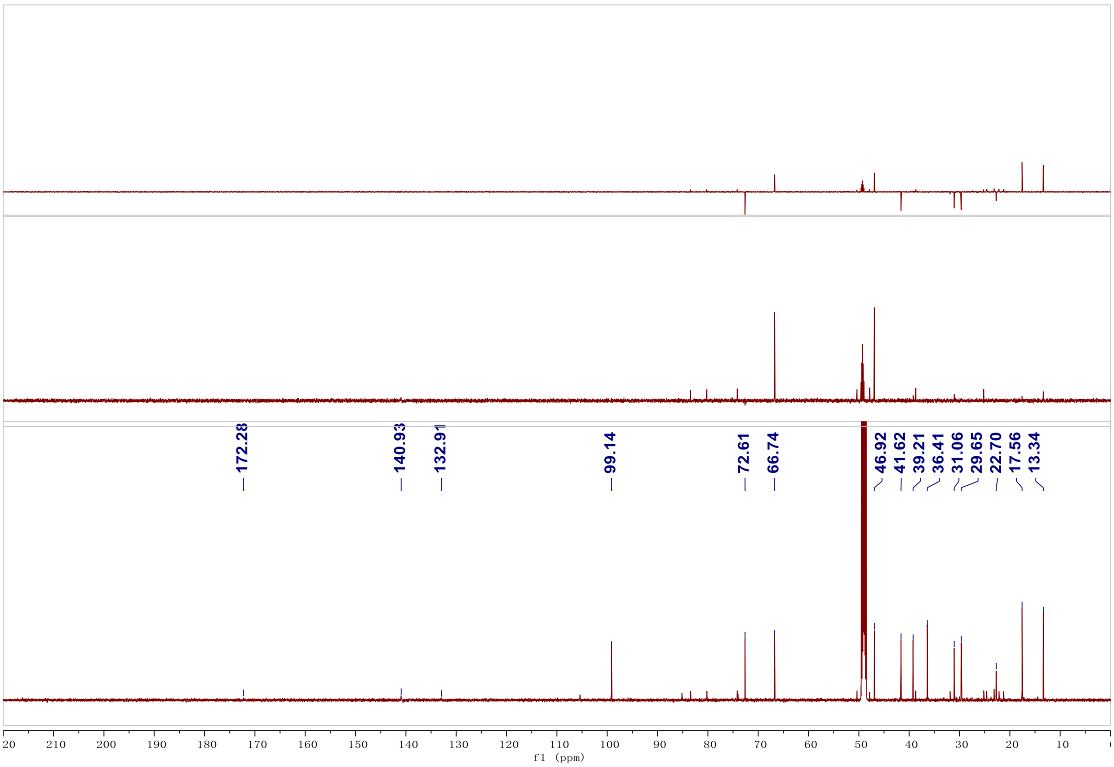

Figure S66. ^{13}C NMR spectrum of compound **8**. (150 MHz, methanol-d_4)

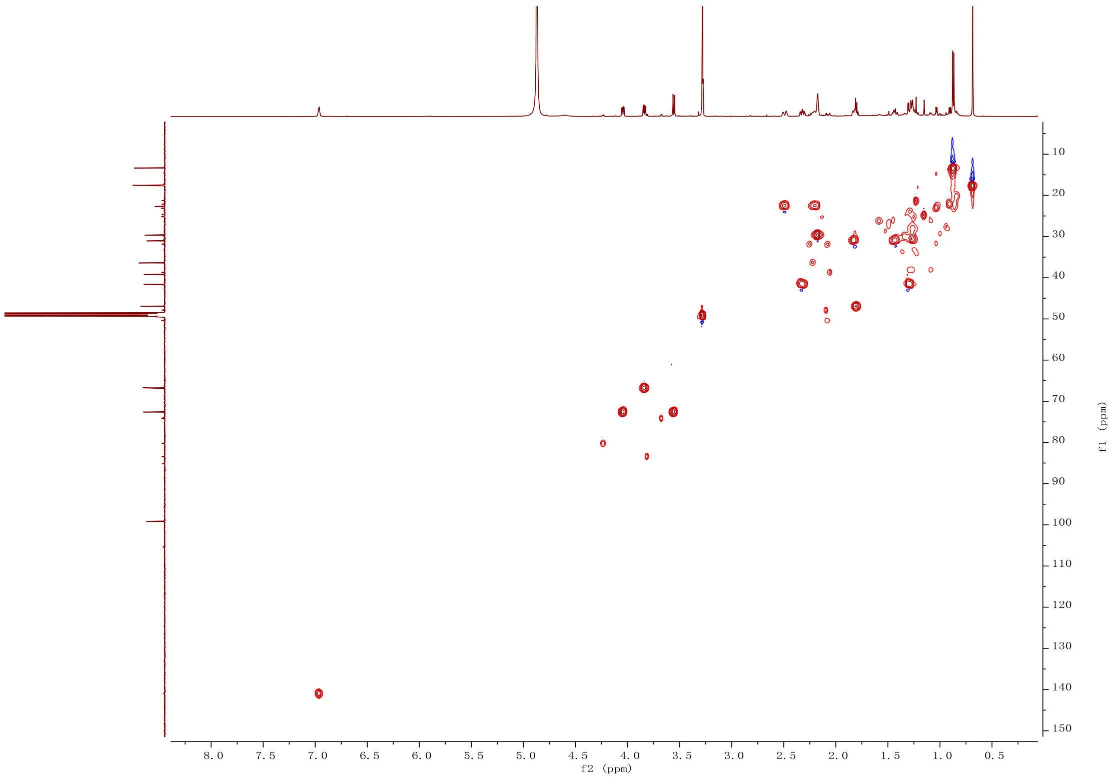

Figure S67. HSQC spectrum of compound **8**

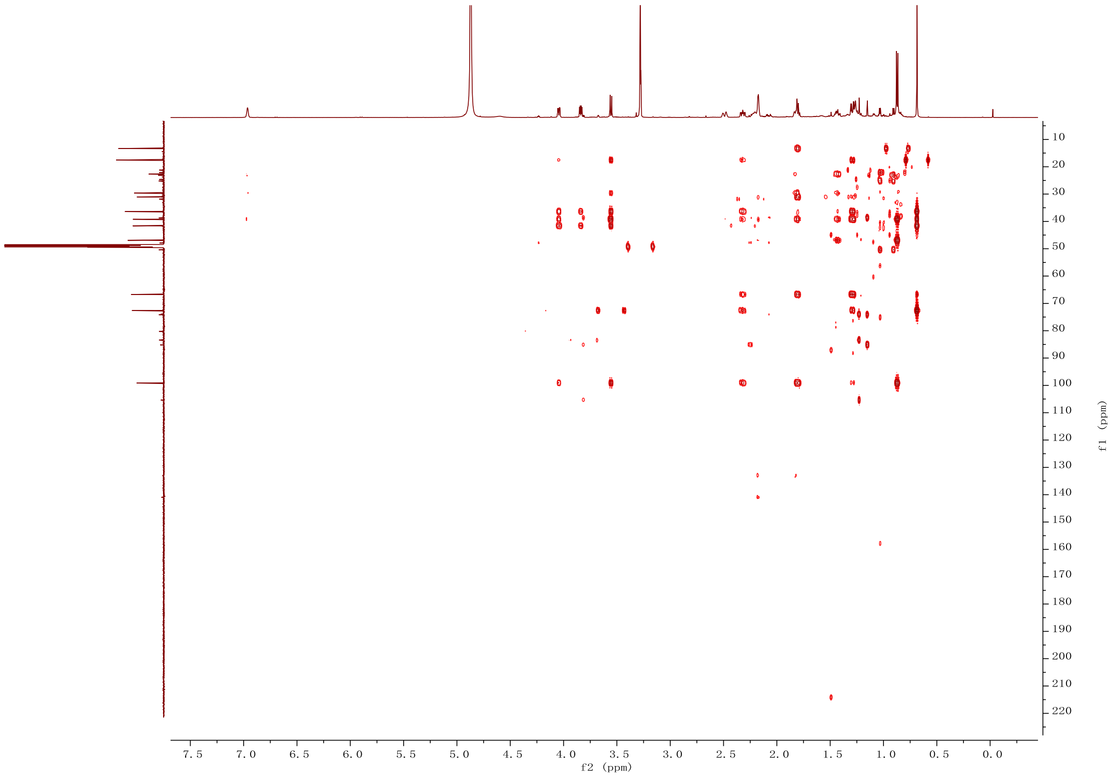

Figure S68. HMBC spectrum of compound **8**

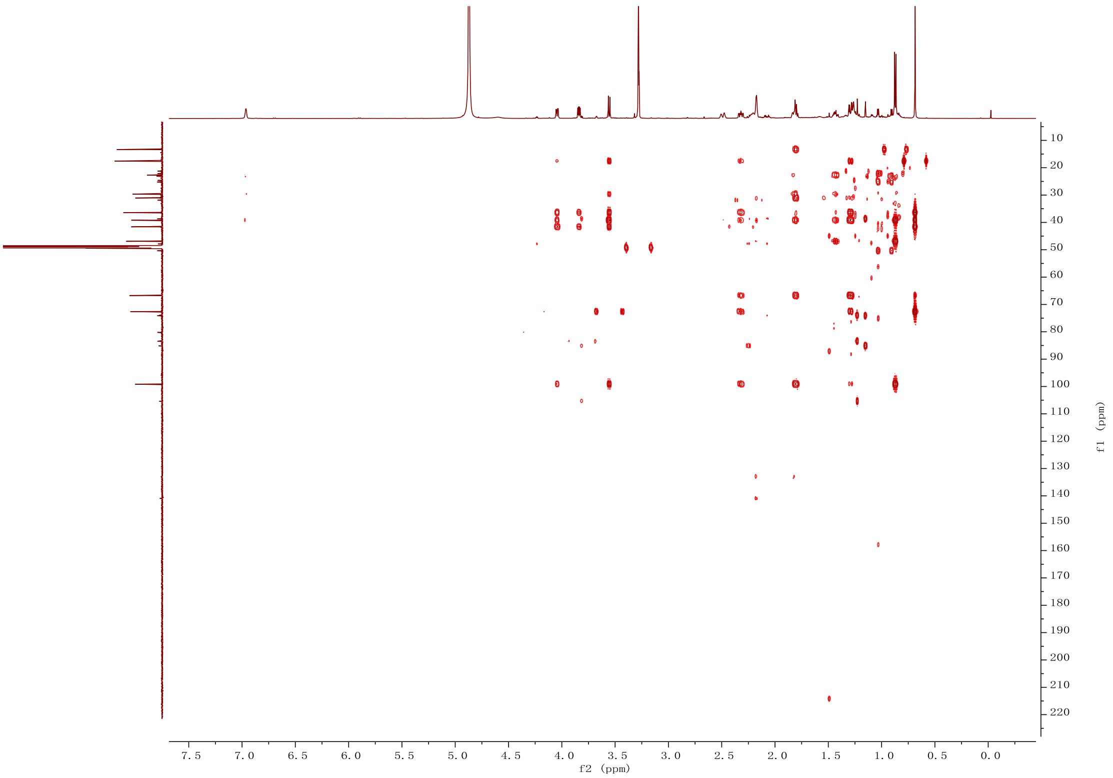

Figure S69. ^{1}H-^{1}H COSY spectrum of compound **8**

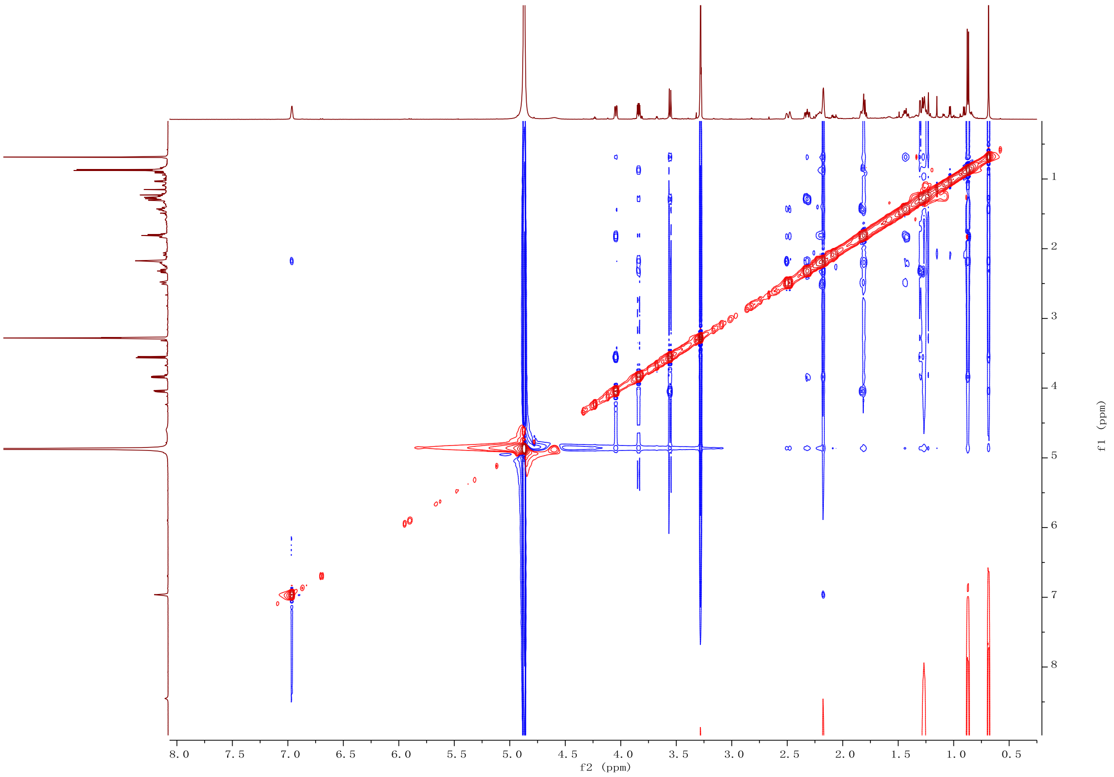

Figure S70. ROESY spectrum of compound **8**

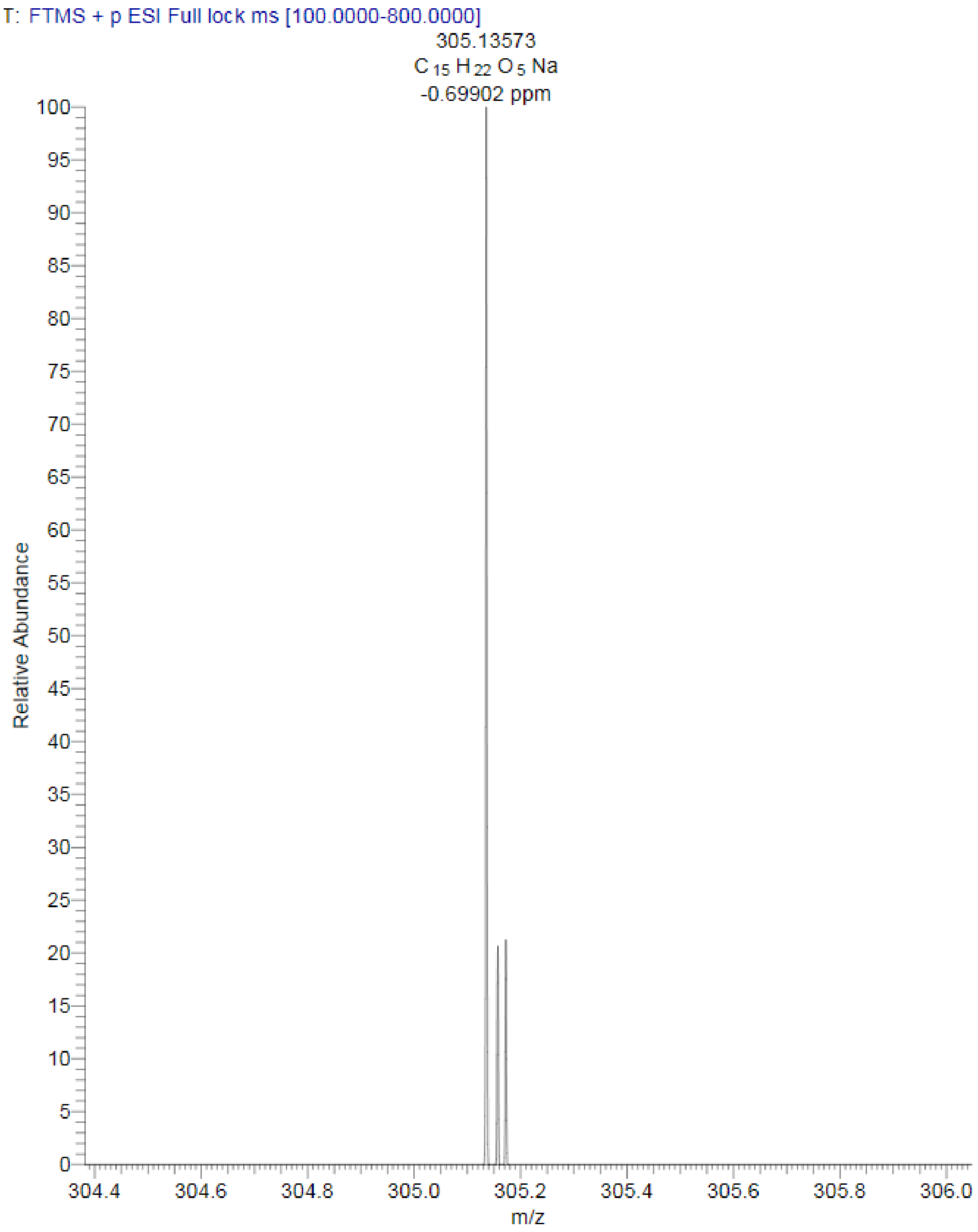

Figure S71. HRESIMS data of compound **8**
